# Pseudaboydins A and B: Novel Isobenzofuranone Derivatives from Marine Fungus *Pseudallescheria boydii* Associated with Starfish *Acanthaster planci*

**DOI:** 10.3390/md12074188

**Published:** 2014-07-14

**Authors:** Wen-Jian Lan, Wei Liu, Wan-Ling Liang, Zeng Xu, Xiu Le, Jun Xu, Chi-Keung Lam, De-Po Yang, Hou-Jin Li, Lai-You Wang

**Affiliations:** 1 School of Pharmaceutical Sciences, Sun Yat-sen University, Guangzhou 510006, China; E-Mails: lanwj@mail.sysu.edu.cn (W.-J.L.); marinewliu@gmail.com (W.L.); natprodlwl@gmail.com (W.-L.L.); lexiu2012@163.com (X.L.); junxu@biochemomes.com (J.X.); lssydp@mail.sysu.edu.cn (D.-P.Y.); 2 Guangdong Technology Research Center for Advanced Chinese Medicine, Guangzhou 510006, China; 3 Institute of Chinese Medical Sciences, Guangdong Pharmaceutical University, Guangzhou 510006, China; 4 School of Chemistry and Chemical Engineering, Sun Yat-sen University, Guangzhou 510275, China; E-Mails: marinezengx@gmail.com (Z.X.); cklam@mail.sysu.edu.cn (C.-K.L.)

**Keywords:** marine fungus, *Pseudallescheria boydii*, isobenzofuranone, pseudaboydins, cytotoxicity

## Abstract

Two novel isobenzofuranone derivatives, pseudaboydins A (**1**) and B (**2**), along with five known compounds, including (*R*)-2-(2-hydroxypropan-2-yl)-2,3-dihydro-5-hydroxybenzofuran (**3**), (*R*)-2-(2-hydroxypropan-2-yl)-2,3-dihydro-5-methoxybenzofuran (**4**), 3,3′-dihydroxy-5,5′-dimethyldiphenyl ether (**5**), 3-(3-methoxy-5-methylphenoxy)-5-methylphenol (**6**) and (−)-regiolone (**7**), were isolated from the culture broth of the marine fungus, *Pseudallescheria boydii*, associated with the starfish, *Acanthaster planci*. Their structures were elucidated primarily based on NMR and MS data. The absolute configurations of **1**–**4** were determined by CD spectroscopy and single-crystal X-ray diffraction studies. The cytotoxic and antibacterial activities of **1**–**4** were evaluated. Pseudaboydin A (**1**) showed moderate cytotoxic activity against human nasopharyngeal carcinoma cell line HONE1, human nasopharyngeal carcinoma cell line SUNE1 and human glandular lung cancer cell line GLC82 with IC_50_ values of 37.1, 46.5 and 87.2 μM, respectively.

## 1. Introduction

**Figure 1:**
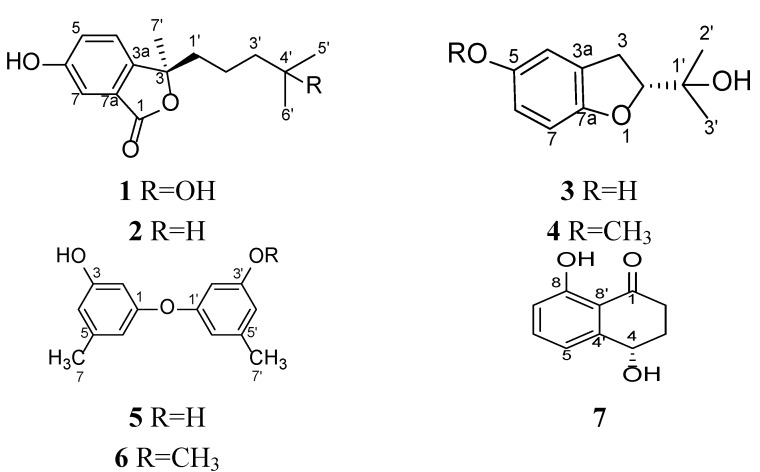
Chemical structures of Compounds **1**–**7**.

In the course of our investigation on the secondary metabolites of marine fungi associated with marine organisms in coral reefs, a variety of novel and/or bioactive metabolites were isolated [1,2,3,4,5,6,7]. The starfish, *Acanthaster planci*, is found easily in the coral reef of the South China Sea and is notorious for preying on corals and is devastating to the coral reef. The arms and upper surface of *Acanthaster planci* are covered by long and sharp spines. If the spines sting a person, it will cause tissue swelling for a week or more. Recently, a fungus was isolated from the inner tissue of *Acanthaster planci*, and it was identified as *Pseudallescheria boydii* based on internal transcribed spacer (ITS) sequence similarity. The marine origin of this fungal species is unprecedented. The terrigenous *Pseudallescheria boydii* is mainly distributed in soil and polluted waters. *Pseudallescheria boydii* is deemed to be a human pathogenic fungus and can cause a variety of infections, especially in the immunocompromised and immunocompetent patients. However, up to now, the literature reports on the secondary metabolites of this fungus are still limited [8,9,10,11,12,13]. The literature survey prompted us to investigate the metabolites of the fungus, *Pseudallescheria boydii*. This fungal strain was cultured in glucose-peptone-yeast extract (GPY) medium. Two new isobenzofuranone derivatives, pseudaboydins A (**1**) and B (**2**), along with five known compounds, (*R*)-2-(2-hydroxypropan-2-yl)-2,3-dihydro-5-hydroxybenzofuran (**3**), (*R*)-2-(2-hydroxypropan-2-yl)-2,3-dihydro-5-methoxybenzofuran (**4**), 3,3′-dihydroxy-5,5′-dimethyldiphenyl ether (**5**), 3-(3-methoxy-5-methylphenoxy)-5-methylphenol (**6**) and (−)-regiolone (**7**), were isolated from the EtOAc extracts of fungal culture broth (Figure 1). Herein, we describe the isolation, structure determination and biological activity evaluation of these compounds.

## 2. Results and Discussion

**Figure 2:**
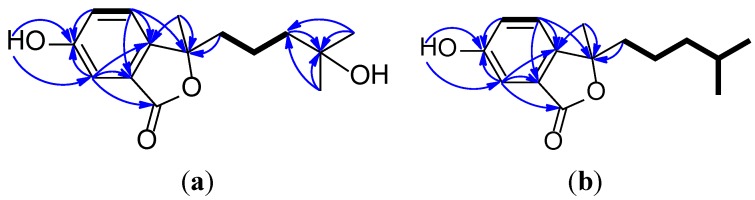
^1^H-^1^H COSY (bold line) and main HMBC (arrow) correlations of **1** and **2** (**a** and **b**).

Pseudaboydin A (**1**) was obtained as a white solid. The molecular formula was determined to be C_15_H_20_O_4_ by HREIMS at *m/z* 264.1355 [M]^+^ (calcd. 264.1362) (Supplementary Figure S2), requiring six degrees of unsaturation. The IR spectrum indicated the presence of hydroxyl groups (3217 cm^−1^), a carbonyl group (1680 cm^−1^) and a benzene ring (3073, 1574 and 1507 cm^−1^). The UV maximum absorption at 217, 243 and 298 nm displayed the carbonyl group conjugated with the benzene ring. The ^13^C-NMR and DEPT spectra displayed three methyls, three methylenes, three methines and six quaternary carbons (Table 1, and Supplementary Figures S4 and S5). The ^1^H-^1^H COSY cross-peaks of H-4/H-5, H_2_-1′/H_2_-2′ and H_2_-2′/H_2_-3′ led to the identification of two isolated proton spin-systems of –CH–CH– and –CH_2_–CH_2_–CH_2_– (Figure 2a, and Supplementary Figure S8). By interpretation of the three aromatic protons at δ_H_ 7.13 (d, *J* = 8.0 Hz, H-4), 7.56 (d, *J* = 8.0 Hz, H-5) and 7.55 (s, H-7) (Supplementary Figure S3), a 1, 2, 4-trisubstituted benzene ring in the molecule was suggested, which was confirmed by HMBC correlations of H-4/C-3a, H-5/C-6, H-7/C-6 and H-7/C-7a (Supplementary Figure S7). The phenolic hydroxy group at δ_H_ 9.33 (brs) showed HMBC correlations with C-5, C-6 and C-7, indicated that it connected to C-6. The HMBC correlations of H-4/C-3 and H-7/C-1 established an isobenzofuranone skeleton. The methylene (H_2_-3′) and two singlet methyl groups at δ_H_ 1.28 and 0.95 (Supplementary Figure S6) showed HMBC correlations with the oxygenated quaternary carbon C-4′ and deduced the only side chain –CH_2_–CH_2_–CH_2_–C(OH)(CH_3_)_2_ in the molecule. This side chain and another methyl group were connected to C-3 based on the HMBC correlations of H_2_-1′/C-3 and H_3_-7′/C-3. 

**Table 1:** ^1^H and ^13^C-NMR data of Compounds **1** and **2** obtained at 400 and 100 MHz, resp., in CDCl_3_, δ in ppm.

Position	1			2		
	δ_C_	Type	δ_H_, Multiple, ( *J* in Hz)	δ_C_	Type	δ_H_, Multiple, ( *J* in Hz)
1	171.8	C		171.6	C	
2						
3	77.7	C		79.1	C	
3a	137.1	C		135.5	C	
4	124.7	CH	7.13, d (8.0)	126.5	CH	7.08, d (8.0)
5	121.5	CH	7.56, d (8.0)	121.3	CH	7.54, d (8.0)
6	157.1	C		156.1	C	
7	119.0	CH	7.55, s	119.3	CH	7.57, s
7a	129.7	C		129.8	C	
1′	33.9	CH_2_	2.45, brd (13.6) 1.65, m	42.9	CH_2_	1.92, dt (14.4, 8.0) 1.80, dt (14.4, 8.0)
2′	16.7	CH_2_	1.71, m	21.7	CH_2_	1.29, m
3′	36.8	CH_2_	1.52, m	39.1	CH_2_	1.16, m
4′	75.4	C		27.8	CH	1.49, nine (6.4)
5′	32.0	CH_3_	1.28, s	22.6	CH_3_	0.82, d (6.4)
6′	24.8	CH_3_	0.95, s	22.4	CH_3_	0.82, d (6.4)
7′	31.4	CH_3_	1.50, s	29.0	CH_3_	1.68, s
6-OH			9.33, brs			9.34, brs
4′-OH			1.80, brs			

**Figure 3:**
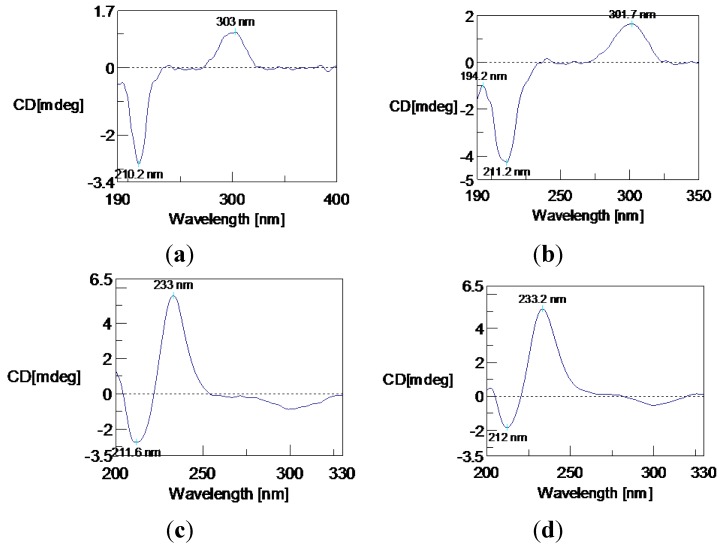
Circular dichroism (CD) spectra of Compounds **1**–**4** (**a**–**d**) in MeCN solution.

The circular dichroism (CD) spectrum of **1** (Figure 3a) exhibited a positive Cotton effect at 303 nm (*n*–π* transition) and a negative Cotton effect at 210.2 nm (π–π* transition). The α, β-unsaturated-γ-lactone core has a planar conformation; Uchida and Kuriyama researched the π–π* CD of α, β-unsaturated-γ-lactones and found the correlation of the Cotton effect with the configuration of the more polarizable bond at the γ-carbon atom [14]. Gawronski *et al.* reported the simple CD method for the determination of the absolute configuration of 5-alkyl and 5-alkoxy-substituted 2(5*H*)-furanones [15]. The chiral center of **1** exists at C-3. The side chain –CH_2_–CH_2_–CH_2_–C(OH)(CH_3_)_2_ makes a greater contribution to negative π–π* and positive *n*–π* Cotton effects than the methyl group. According to Uchida and Gawronski’s empirical rules, the γ-carbon atom (C-3) was assigned to be *R* configuration. Therefore, Compound **1** was determined as (*R*)-3-methyl-3-(4-hydroxy-4-methylpentyl)-6-hydroxy isobenzofuran-1(3*H*)-one.

Pseudaboydin B (**2**) was isolated as a white solid. The molecular formula of **2** was established as C_15_H_20_O_3_ on the basis of HREIMS (*m*/*z* 248.1408 [M]^+^, calcd. 248.1412) (Supplementary Figure S10) and NMR data (Table 1, and Supplementary Figures S11–S16). The general features of its UV, IR and NMR spectra data were very similar to those of **1**. The ^13^C-NMR and DEPT spectra displayed three methyls, three methylenes, four methines and five quaternary carbons. A quick identification was made that oxygenated quaternary carbon C-4′ was replaced by a methine. Two methyl doublets at δ_H_ 0.82 (d, *J* = 6.4 Hz, H_3_-5′ and H_3_-6′) connected to the methine at δ_H_ 1.49 (nine, *J* = 6.4 Hz, H-4′). The ^1^H-^1^H COSY cross-peaks of H_2_-1′/H_2_-2′, H_2_-2′/H_2_-3′, H_2_-3′/H-4′, H-4′/H_3_-5′ and H-4′/H_3_-6′ also confirmed the presence of the aliphatic side chain 4-methylpentyl (Figure 2b). By careful analysis of HMQC, ^1^H-^1^H COSY and HMBC spectra data, Compound **2** was assigned as 3-methyl-3-(4-methylpentyl)-6-hydroxyisobenzofuran-1(3*H*)-one. The stereochemistry of Compound **2** was also established by CD spectrum (Figure 3b). **2** showed a very similar CD spectrum with that of **1**. Thus, the absolute configuration of **2** was also definitely assigned as 3*R*. 

**Figure 4:**
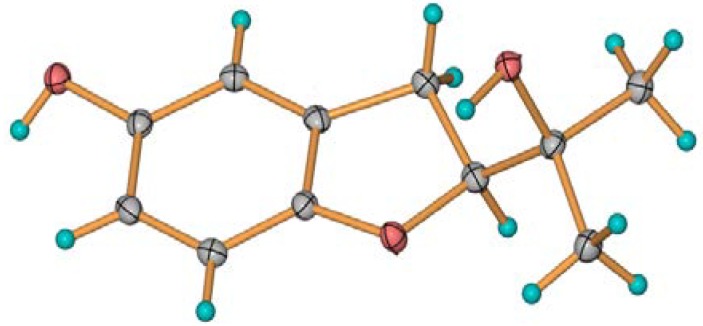
Crystal structure of **3**. Thermal ellipsoids are plotted at a 30% probability level.

Compounds **3** and **4** were identified as 2-(2-hydroxypropan-2-yl)-2,3-dihydro-5-hydroxybenzofuran (**3**) and 2-(2-hydroxypropan-2-yl)-2,3-dihydro-5-methoxybenzofuran (**4**). Compound **3** was previously isolated from the fungus, *Acremonium murorum* [16]; **4** was obtained by synthesis [17]. The NMR data of **3** recorded in DMSO-d6 (Table 2, and Supplementary Figures S17 and S18) were slightly different from the reference data recorded in CD_3_OD, and the ^13^C-NMR data (Supplementary Figures S19 and S20) of **4** were not reported previously. Furthermore, the absolute configurations of their chiral centers were not yet reported. Fortunately, we obtained a single crystal of **3** from the MeOH solution. In the crystal, the molecules are arranged in a helical manner and connected by strong O-H...O hydrogen bonds. The helicates are further joined together by O-H...O hydrogen bonds to form a discrete molecular layer with hydrophobic substituents pointing outwards. With this kind of layer-type arrangement, the crystal structure is stabilized by the weak van der Waals’ interactions. The absolute configuration of **3** was determined as 2*R* by analysis of the single-crystal X-ray data (Figure 4). The Flack absolute structure parameter is 0.2 (4) [18]. In the CD spectrum, **3** showed a positive Cotton effect at 233 nm and a negative Cotton effect at 211.6 nm (Figure 3c). Finally, the CD spectrum of **4** (Figure 3d) was almost identical with that of **3**. Thus, the absolute configuration of **4** was also determined as 2*R*.

**Table 2:** ^1^H and ^13^C-NMR data of Compounds **3** and **4** obtained at 400 and 100 MHz, resp., δ in ppm.

Position	3 ^a^			4 ^b^		
	δ_C_	Type	δ_H_, Multiple, (*J* in Hz)	δ_C_	Type	δ_H_, Multiple, (*J* in Hz)
1						
2	88.6	CH	4.42, dd (9.6, 8.4)	89.4	CH	4.58, dd (9.2, 8.8)
3	30.5	CH_2_	3.06, dd (16.0, 8.4); 2.98, dd (16.0, 9.6)	31.2	CH_2_	3.17, dd (16.0, 8.8); 3.10, dd (16.0, 9.2)
3a	128.1	C		128.1	C	
4	111.9	CH	6.59, d (2.4)	111.2	CH	6.75, d (1.6)
5	150.9	C		154.1	C	
6	113.3	CH	6.42, dd (8.4, 2.4)	112.8	CH	6.64, dd (8.8, 1.6)
7	108.3	CH	6.50, d (8.4)	108.9	CH	6.68, d (8.8)
7a	152.5	C		153.7	C	
1′	70.1	C		71.8	C	
2′	24.7	CH_3_	1.09, s	23.9	CH_3_	1.20, s
3′	26.2	CH_3_	1.11, s	26.2	CH_3_	1.33, s
5-OH			8.69, s			
1′-OH			4.50, s			1.97, brs
5-OCH_3_				56.0	CH_3_	3.75, s

^a^ Measured in DMSO-*d*_6_ and DMSO-*d*_6_ was used as an internal standard (δ_C_ 39.51, δ_H_ 2.50); ^b^ measured in CDCl_3_ and CDCl_3_ was used as an internal standard (δ_C_ 77.0, δ_H_ 7.26).

Compounds **5**–**7** were determined as 3,3′-dihydroxy-5,5′-dimethyldiphenyl ether [19], 3-(3-methoxy-5-methylphenoxy)-5-methyl phenol [20] and (−)-regiolone [21] respectively, by comparing their spectroscopic data (Supplementary Figures S21–S28) with the literature values. 

Compounds **1**–**4** were evaluated for *in vitro* cytotoxicity against human nasopharyngeal carcinoma cell line HONE1, human nasopharyngeal carcinoma cell line SUNE1 and human glandular lung cancer cell line GLC82. As a result, Compound **1** showed moderate cytotoxic activity against HONE1, SUNE1 and GLC82 with IC_50_ values of 37.1, 46.5 and 87.2 μM, respectively. Compounds **2**–**4** displayed no cytotoxicity against the tested cancer cell lines (IC_50_ > 100 μM). Additionally, Compounds **1**–**4** were also evaluated for their antibacterial activity against MRSAST239, standard *Staphylococcus aureus* (ATCC29213) and *Escherichia coli* (ATCC295225), using a broth dilution method (Mueller–Hinton broth). Vancomycin and ampicillin sodium were used as positive control. However, Compounds **1**–**4** did not exhibit significant activity at a concentration lower than 256 μg/mL. 

## 3. Experimental Section

### 3.1. General Experimental Procedures

Preparative HPLC was performed using a Shimadzu LC-20AT HPLC pump (Shimadzu Corporation, Nakagyo-ku, Kyoto, Japan) equipped with an SPD-20A dual λ absorbance detector (Shimadzu Corporation, Nakagyo-ku, Kyoto, Japan) and a Shim-pack PRC-ODS HPLC column (250 mm × 20 mm, Shimadzu Corporation, Nakagyo-ku, Kyoto, Japan). Optical rotations were measured using a Schmidt and Haensch Polartronic HNQW5 optical rotation spectrometer (SCHMIDT + HAENSCH GmbH & Co., Berlin, Germany). CD spectra were measured on a JASCO J-810 circular dichroism spectrometer (JASCO International Co. Ltd., Hachioji, Tokyo, Japan). IR spectra were recorded on a PerkinElmer Frontier FT-IR spectrophotometer (PerkinElmer Inc., Waltham, MA, USA). UV spectra were recorded on a Shimadzu UV-Vis-NIR spectrophotometer (Shimadzu Corporation, Nakagyo-ku, Kyoto, Japan). 1D and 2D NMR spectra were recorded on a Bruker Avance II 400 spectrometer (Bruker BioSpin AG, Industriestrasse 26, Fällanden, Switzerland). The chemical shifts are relative to the residual solvent signals (CDCl_3_: δ_H_ 7.26 and δ_C_ 77.0; CD_3_OD: δ_H_ 3.31 and δ_C_ 49.15; DMSO-*d*_6_: δ_H_ 2.50 and δ_C_ 39.51). Mass spectra were obtained on a Thermo DSQ EI low-resolution mass spectrometer (Thermo Fisher Scientific, Waltham, MA, USA) and a Thermo MAT95XP EI high-resolution mass spectrometer (Thermo Fisher Scientific, Waltham, MA, USA). X-ray diffraction data were acquired on a Bruker SMART APEX CCD X-ray single-crystal diffractometer (Bruker AXS GmbH, Karlsruhe, Germany).

### 3.2. Fungal Strain and Culture Method

Marine fungus *Pseudallescheria boydii* was isolated from the inner tissue of sea star *Acanthaster planci* collected from Hainan Sanya National Coral Reef Reserve, Hainan, China. A voucher specimen was deposited in the School of Pharmaceutical Sciences, Sun Yat-sen University, Guangzhou, China.

The fermentation medium was glucose 10 g/L, peptone 5 g/L, yeast extract 2 g/L, sea water 1 L and pH 7.5 (GPY medium). Fungal mycelia were cut and transferred aseptically to 500-mL Erlenmeyer flasks, each containing 200 mL sterilized GPY liquid medium. The flasks were incubated at 28 °C on a rotary shaker (120 rpm) for 20 days.

### 3.3. Extraction and Isolation

Eighty liters of liquid culture were filtered through cheesecloth. The culture broth was extracted three times with EtOAc (80 L) and then was concentrated by low-temperature rotary evaporation to give an extract (30.9 g). The extract was subjected to column chromatography (diameter: 8 cm, length: 70 cm) over silica gel (200 g) eluting with petroleum ether (2 L)-EtOAc (2 L) (100:0–0:100) followed by EtOAc (2 L)-MeOH (2 L) (100:0–0:100) to yield 15 fractions (code: Fr. 1–Fr. 15). Fr. 8 (1.08 g) was further separated by RP-HPLC (MeCN-H_2_O, 4:6) to afford Compounds **1** (18 mg) and **2** (26 mg). Fr. 12 (3.22 g) was further separated by RP-HPLC (MeOH-H_2_O, 4:6) to afford Compounds **3** (1608 mg) and **4** (50 mg). Fr. 5 (1.58 g) was purified by RP-HPLC (MeOH-H_2_O, 6:4) to give Compounds **5** (65 mg), **6** (18 mg) and **7** (12 mg).

Pseudaboydin A ((*R*)-3-methyl-3-(4-hydroxy-4-methylpentyl)-6-hydroxyisobenzofuran-1(3*H*)-one, (**1**): white solid. 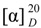: +200° (*c* = 0.025, MeOH). CD (MeCN) λ_max_ (Δε): 210.2 (−5.41), 303 (+2.01) nm. IR (KBr): 3217, 3073, 2978, 1680, 1574, 1507, 1419, 1389, 1354, 1287, 1230, 1161, 1065, 957, 891, 763 cm^−1^. UV (MeOH) λ_max_ (ε): 217 nm (8152), 243 nm (7142*)*, 298 nm (2247*)*. ^1^H and ^13^C-NMR: Table 1. LREIMS: *m/z* 264, 246, 231, 203, 189, 181, 165, 147, 133, 119, 105, 91, 69. HREIMS: *m/z* 264.1355 [M]^+^ (calcd. for C_15_H_20_O_4_^+^: 264.1362). 

Pseudaboydin B ((*R*)-3-methyl-3-(4-methylpentyl)-6-hydroxyisobenzofuran-1(3*H*)-one, (**2**): white solid. 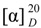: +53.6° (*c =* 0.078, MeOH). CD (MeCN) λ_max_ (Δε): 211.2 (−7.22), 301.7 (+2.78) nm. IR (KBr): 3377, 3073, 2952, 1687, 1575, 1510, 1467, 1380, 1294, 1223, 1127, 1052, 947, 890, 766 cm^−1^. UV (MeOH) λ_max_ (ε): 219 nm (11,833), 244 nm (12,258), 299 nm (4202). ^1^H and ^13^C-NMR: Table 1. LREIMS: *m/z* 248, 233, 203, 192, 178, 165, 147, 133, 119, 105, 91, 77. HREIMS: *m/z* 248.1408 [M]^+^ (calcd. for C_15_H_20_O_3_^+^: 248.1412). 

(*R*)-2-(2-hydroxypropan-2-yl)-2,3-dihydro-5-hydroxybenzofuran (**3**): colorless solid. 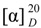: +81.3° (*c* = 0.49, MeOH). CD (MeCN) λ_max_ (Δε): 211.6 (−3.21), 233 (+6.53) nm. ^1^H and ^13^C-NMR: Table 2. LREIMS: *m/z* 194, 176, 161, 136, 123, 107, 77, 59.

(*R*)-2-(2-hydroxypropan-2-yl)-2,3-dihydro-5-methoxybenzofuran (**4**): colorless oil. 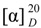: +42.5° (*c* = 0.41, MeOH). CD (MeCN) λ_max_ (Δε): 212 (−2.32), 233.2 (+6.48) nm. ^1^H and ^13^C-NMR: Table 2. LREIMS: *m/z* 208, 190, 175, 150, 135, 121, 107, 91, 77. 

3,3′-Dihydroxy-5,5′-dimethyldiphenyl ether (**5**): colorless oil. ^13^C-NMR (CDCl_3_, 100 MHz) δ_C_: 158.5 (C-1, 1′), 157.6 (C-3, 3′), 140.1 (C-5, 5′), 111.2 (C-6, 6′), 110.1 (C-4, 4′), 103.0 (C-2, 2′), 21.1 (C-7, 7′). ^1^H NMR (CDCl_3_, 400 MHz) δ_H_: 9.44 (brs, 3-OH, 3′-OH), 6.35 (brs, H-6, 6′), 6.25 (brs, H-4, 4′), 6.17 (brs, H-2, 2′), 2.18 (s, H-7, 7′).

3-(3-Methoxy-5-methylphenoxy)-5-methylphenol (**6**): colorless oil. ^13^C-NMR (CDCl_3_, 100 MHz) δ_C_: 160.6 (C), 158.3 (C), 157.8 (C), 156.5 (C), 140.9 (C), 140.6 (C), 112.2 (CH), 111.9 (CH), 110.9 (CH), 110.0 (CH), 103.2 (CH), 102.3 (CH), 55.3 (CH_3_), 21.6 (CH_3_), 21.4 (CH_3_). ^1^H NMR (CDCl_3_, 400 MHz) δ_H_: 6.49 (s, 1H), 6.43 (s, 1H), 6.41 (s, 1H), 6.39 (s, 2H), 6.30 (s, 1H), 4.91 (brs, OH), 3.76 (s, OCH_3_), 2.29 (s, CH_3_), 2.26 (s, CH_3_). ^13^C-NMR (CD_3_OD, 100 MHz) δ_C_: 162.4 (C), 159.8 (C), 159.72 (C), 159.70 (C), 141.8 (2 × C), 112.9 (CH), 112.2 (CH), 111.9 (CH), 110.7 (CH), 104.4 (CH), 103.2 (CH), 55.9 (CH_3_), 21.8 (CH_3_), 21.7 (CH_3_). ^1^H NMR (CD_3_OD, 400 MHz) δ_H_: 6.46 (s, 1H), 6.35 (s, 2H), 6.30 (s, 1H), 6.25 (s, 1H), 6.18 (s, 1H), 3.70 (s, OCH_3_), 2.24 (s, CH_3_), 2.20 (s, CH_3_). LREIMS: *m/z* 244, 229, 213, 201, 183, 173, 169, 158, 145, 122, 115.

(−)-Regiolone (**7**): colorless oil. 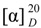: −9.6° (*c* = 0.12, MeOH). ^13^C-NMR (CDCl_3_, 100 MHz) δ_C_: 204.3 (C-1), 162.7 (C-8), 145.9 (C-4′), 137.0 (C-6), 117.8 (C-7), 117.4 (C-5), 115.2 (C-8′), 67.7 (C-4), 34.6 (C-2), 31.2 (C-3). ^1^H NMR (CDCl_3_, 400 MHz) δ_H_: 12.41 (brs, 8-OH), 7.49 (dd, *J* = 8.4, 7.2 Hz, H-6), 7.02 (d, *J* = 7.2 Hz, H-5), 6.92 (d, *J* = 8.4 Hz, H-7), 4.91 (dd, *J* = 8.0, 4.0 Hz, H-4), 3.00 (ddd, *J* = 17.6, 8.4, 4.8 Hz, H-2a), 2.64 (ddd, *J* = 17.6, 8.0, 4.8 Hz, H-2b), 2.34 (dddd, *J* = 15.2, 8.4, 4.8, 4.0 Hz, H-3a), 2.19 (dddd, *J* = 15.2, 8.0, 8.0, 4.8 Hz, H-2b), 1.97 (s, 4-OH). LREIMS: *m/z* 178, 160, 150, 132, 121, 104, 93, 77, 65, 51.

### 3.4. Crystal Structure Determination of 3

A crystal of **3** was obtained from MeOH solution. C_11_H_14_O_3_, *M* = 194.22, colorless block, orthorhombic system, space group *P*2(1)2(1)2(1), *a* = 6.2067(3), *b* = 9.1962(3), *c* = 16.9308(7) Å. *V* = 966.37(7) Å^3^. *Z* = 4, *d* = 1.335 g/cm^3^, crystal size 0.40 × 0.39 × 0.41 mm^3^. The absolute flack parameter is 0.2 (4). X-ray diffraction data were collected on a Bruker SMART APEX CCD diffractometer with Cu *K_α_* radiation (λ = 1.54184 Å) at a temperature of 293 K. The data were processed using CrysAlis. The structures were solved by a direct method. H-atoms were added in ideal positions and refined as riding models. The structures were refined using full-matrix least-squares based on *F*2 with the program, SHELXL [22,23]. 

The Cambridge Crystallographic Data Centre (CCDC) 1004603 contains the supplementary crystallographic data of Compound **3** [24].

### 3.5. Cytotoxicity Assay

The *in vitro* cytotoxicities of **1**–**4** were assayed using a standard protocol [1,2,3]. Hirsutanol A, a potent anticancer agent isolated from marine fungal metabolites, was used as a positive control, and it showed cytotoxic activities against the tested cancer cell lines, HONE1, SUNE1 and GLC82, with IC_50_ values of 17.4, 3.5 and 10.1 μM, respectively [4].

### 3.6. Antibacterial Activity Assay

Vancomycin and ampicillin sodium were used as a positive control. The MIC values were determined using a broth dilution method (Mueller–Hinton broth) based on the National Committee on Clinical Laboratory Standards (NCCLS). The starting concentrations of the tested compounds were 256 µg/mL (from 256 to 0.25). The solution of compound in DMSO (10 µL) was added to 90 µL of bacterial culture (1 × 106 CFU/mL) in the first well of flat-bottomed 96-well tissue culture plates. The solution was then double diluted. The bacterial culture solution containing the appropriate compound (50 µL) was discarded from the last well in order to ensure 100-µL volume of bacterial culture in every well. A set of tubes containing only inoculated broth was kept as a control. The plate was incubated at 37 °C overnight in an electro-heating standing-temperature cultivator before the measurement of the absorbance value. The optical density values at 600 nm were measured using a multifunction microplate reader (PowerWaveTM XS2, BioTek^®^ Instruments Inc., Winooski, VT, USA). 

## 4. Conclusions

Isobenzofuranones, fused-ring aromatic γ-lactones, showed various biological activities and have attracted much attention from synthetic chemists [25]. However, naturally occurring isobenzofuranones are few and mainly found in plants and endophytic fungi [26,27,28,29,30]. Moreover, the absolute configurations at the C-3 position of many substituted isobenzofuranone still remain unknown. Pseudaboydins A (**1**) and B (**2**) are unprecedented isobenzofuranone derivatives with a methylpentyl side chain. The absolute configurations of **1** and **2** were unambiguously determined as 3*R* by CD spectra analysis. The stereochemistry of Compound **3** was first elucidated as 2*R* by single-crystal X-ray diffraction analysis. Compound **4** was also assigned as having 2*R* absolute configuration for the CD, similar to **3**. 

## Supplementary Files

Supplementary File 1 Supplementary Information (PDF, 2391 KB)
