# Supplementary material for: Pseudaboydins A and B: Novel Isobenzofuranone Derivatives from Marine Fungus Pseudallescheria boydii Associated with Starfish Acanthaster planci
Source: Mar Drugs. 2014 Jul 14;12(7):4188–99. doi: 10.3390/md12074188 (PMC4113822; doi:10.3390/md12074188)
Supplement: Supplementary File 1 — Supplementary Information (PDF, 2391 KB) [file marinedrugs-12-04188-s001.pdf]

Supplementary Information

Figure S1. LREIMS of pseudaboydin A (**1**).
Figure S2. HREIMS of pseudaboydin A (**1**).
Figure S3. ^{1}H-NMR (400 MHz, $CDCl_3$) spectrum of pseudaboydin A (**1**).
Figure S4. ^{13}C-NMR (100 MHz, $CDCl_3$) spectrum of pseudaboydin A (**1**).
Figure S5. DEPT135 and DEPT90 of pseudaboydin A (**1**).
Figure S6. gHMQC of pseudaboydin A (**1**).
Figure S7. gHMBC of pseudaboydin A (**1**).
Figure S8. ^{1}H-^{1}H gCOSY of pseudaboydin A (**1**).
Figure S9. LREIMS of pseudaboydin B (**2**).
Figure S10. HREIMS of pseudaboydin B (**2**).
Figure S11. ^{1}H-NMR (400 MHz, $CDCl_3$) spectrum of pseudaboydin B (**2**).
Figure S12. ^{13}C-NMR (100 MHz, $CDCl_3$) spectrum of pseudaboydin B (**2**).
Figure S13. DEPT135 and DEPT90 of pseudaboydin B (**2**).
Figure S14. gHMQC of pseudaboydin B (**2**).
Figure S15. gHMBC of pseudaboydin B (**2**).
Figure S16. ^{1}H–^{1}H gCOSY of pseudaboydin B (**2**).
Figure S17. ^{1}H-NMR (400 MHz, DMSO-d6) spectrum of (*R*)-2-(2-hydroxypropan-2-yl)-2, 3-dihydro-5-hydroxybenzofuran (**3**).
Figure S18. ^{13}C-NMR (100 MHz, DMSO-d6) spectrum of (*R*)-2-(2-hydroxypropan-2-yl)-2, 3-dihydro-5-hydroxybenzofuran (**3**).
Figure S19. ^{1}H-NMR (400 MHz, $CDCl_3$) spectrum of (*R*)-2-(2-hydroxypropan-2-yl)-2, 3-dihydro-5-methoxybenzofuran (**4**).
Figure S20. ^{13}C-NMR (100 MHz, $CDCl_3$) spectrum of (*R*)-2-(2-hydroxypropan-2-yl)-2, 3-dihydro-5-methoxybenzofuran (**4**).
Figure S21. ^{1}H-NMR (400 MHz, $CDCl_3$) spectrum of 3,3′-dihydroxyl-5,5′-dimethyldiphenyl ether (**5**).
Figure S22. ^{13}C-NMR (100 MHz, $CDCl_3$) spectrum of 3,3′-dihydroxyl-5,5′-dimethyldiphenyl ether (**5**).
Figure S23. ^{1}H-NMR (400 MHz, $CDCl_3$) spectrum of 3-(3-methoxy-5-methyl phenoxy)-5-methylphenol (**6**).
Figure S24. ^{13}C-NMR (100 MHz, $CDCl_3$) spectrum of 3-(3-methoxy-5-methyl phenoxy)-5-methylphenol (**6**).
Figure S25. ^{1}H-NMR (400 MHz, CD_3OD) spectrum of 3-(3-methoxy-5-methyl phenoxy)-5-methylphenol (**6**).
Figure S26. ^{13}C-NMR (100 MHz, CD_3OD) spectrum of 3-(3-methoxy-5-methyl phenoxy)-5-methylphenol (**6**).
Figure S27. ^{1}H-NMR (400 MHz, $CDCl_3$) spectrum of (−)-regiolone (**7**).
Figure S28. ^{13}C-NMR (100 MHz, $CDCl_3$) spectrum of (−)-regiolone (**7**).

Figure S1. LREIMS of pseudaboydin A (**1**).

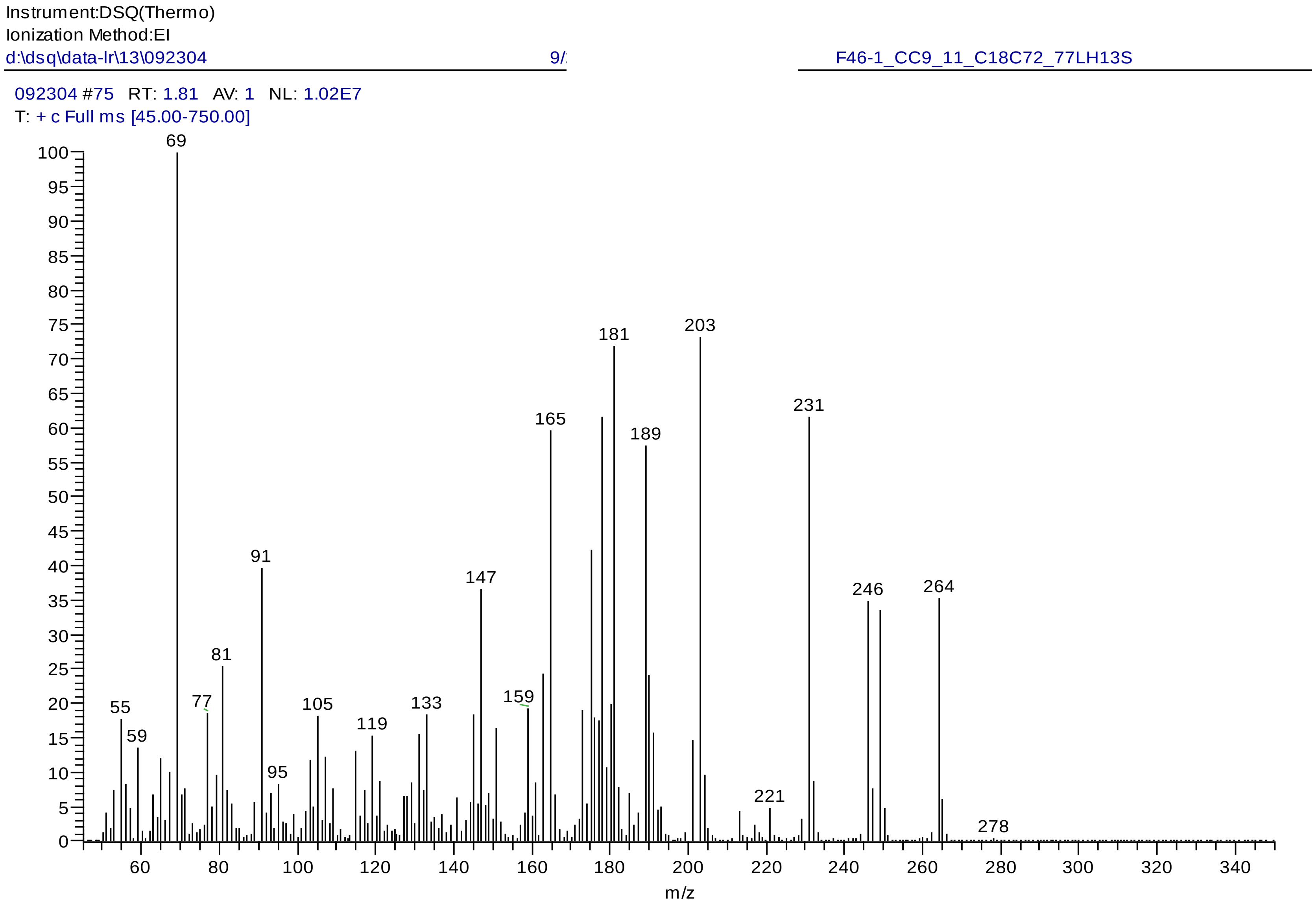

Figure S2. HREIMS of pseudaboydin A (**1**).

SPECTRUM—MS
File: D:\DATA-HR\13\092603-f46-c1.RAW
Full ms [251.500–272.500]—Range: 264.000–264.500
Scan No. 13 of 21
Scan #: 13
RT: 0.51
Data points: 1

Mass	Relative Intensity	Theoretical Mass	Delta [ppm]	Delta [mmu]	RDB	Composition
264.1355	47.0	264.1356	-0.4	-0.1	6.0	$C_{15}H_{20}O_4$

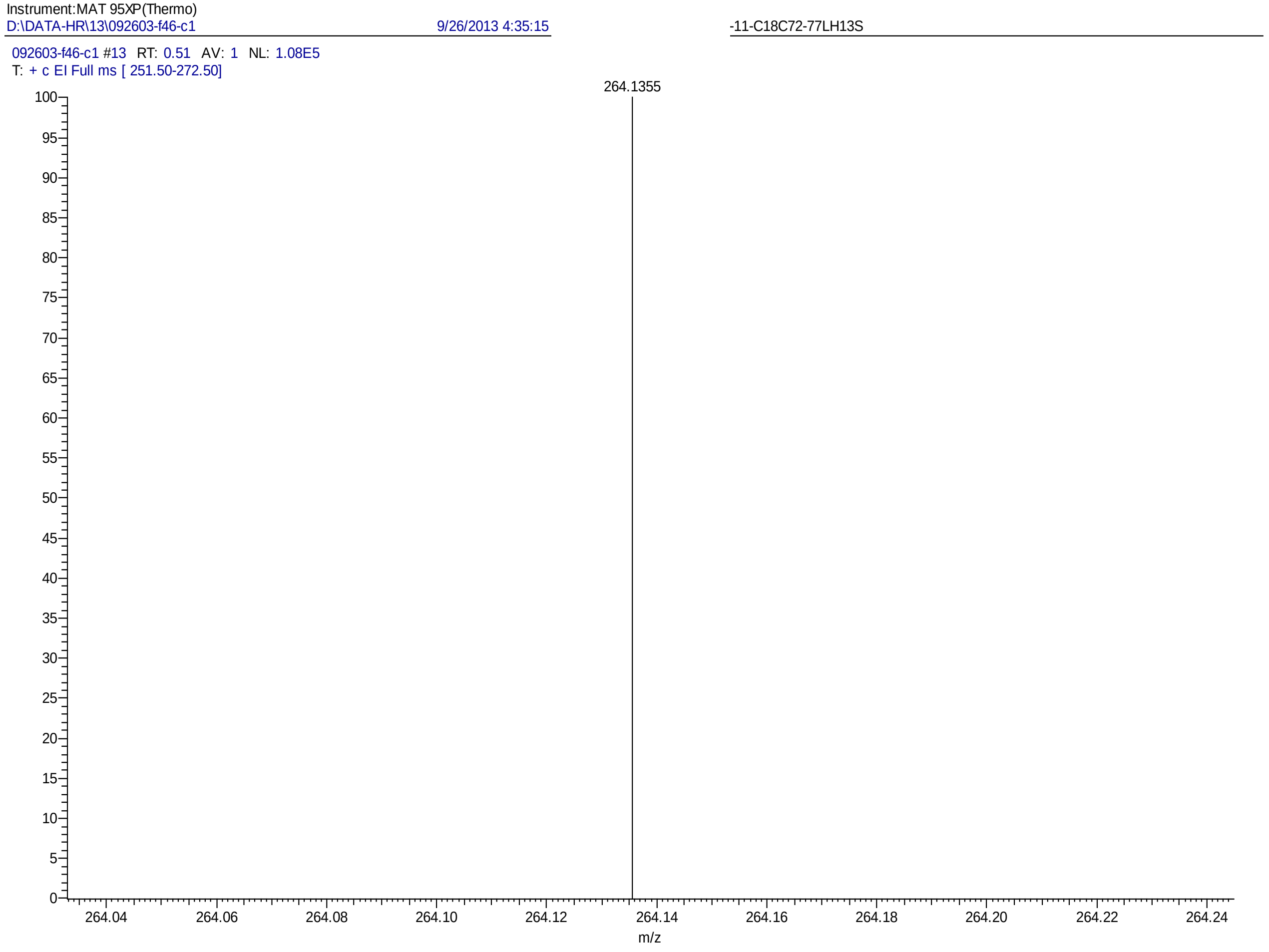

Figure S3. ^{1}H-NMR (400 MHz, $CDCl_3$) spectrum of pseudaboydin A (**1**).

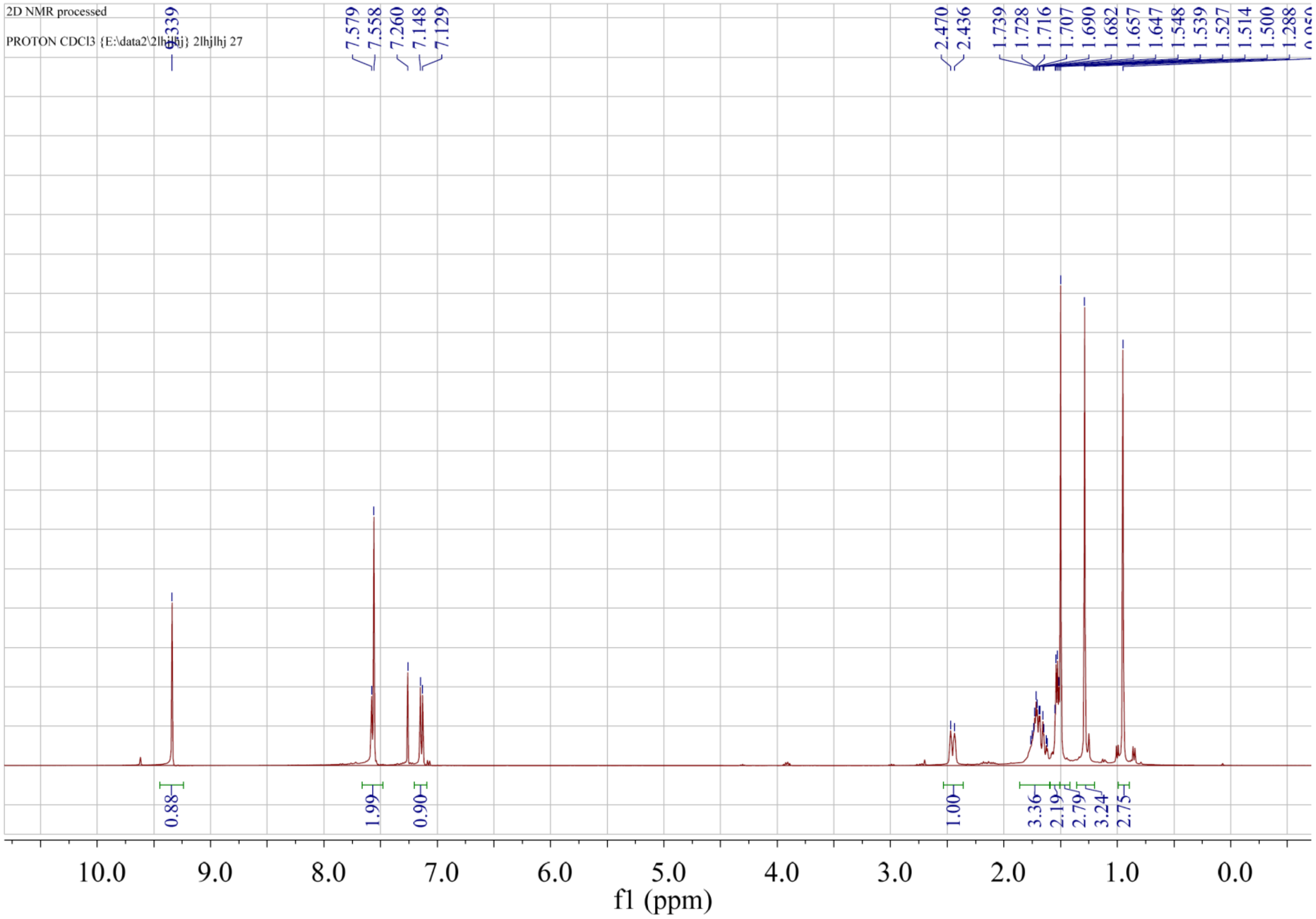

Figure S4. ^{13}C-NMR (100 MHz, $CDCl_3$) spectrum of pseudaboydin A (**1**).

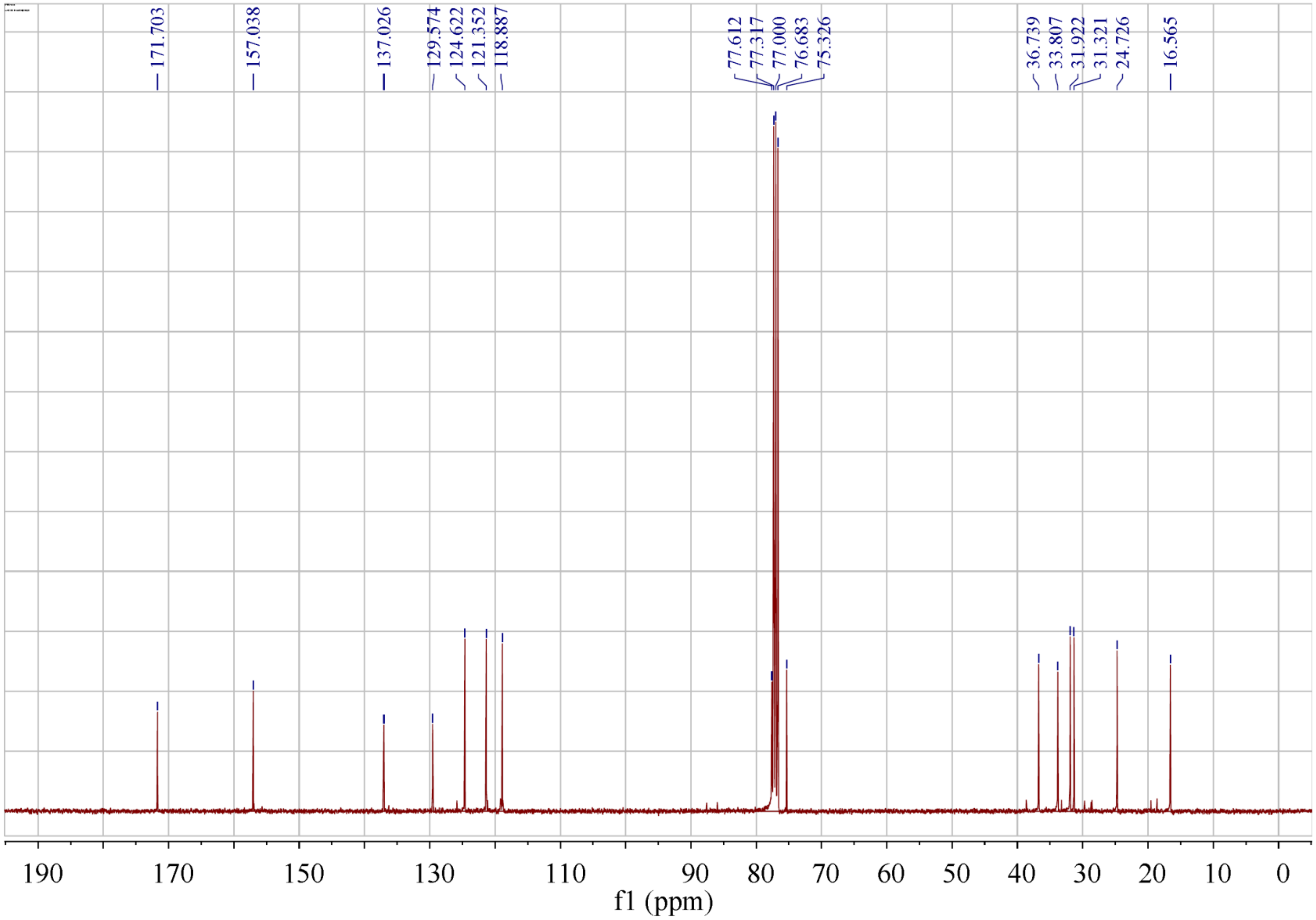

Figure S5. DEPT135 and DEPT90 of pseudaboydin A (**1**).

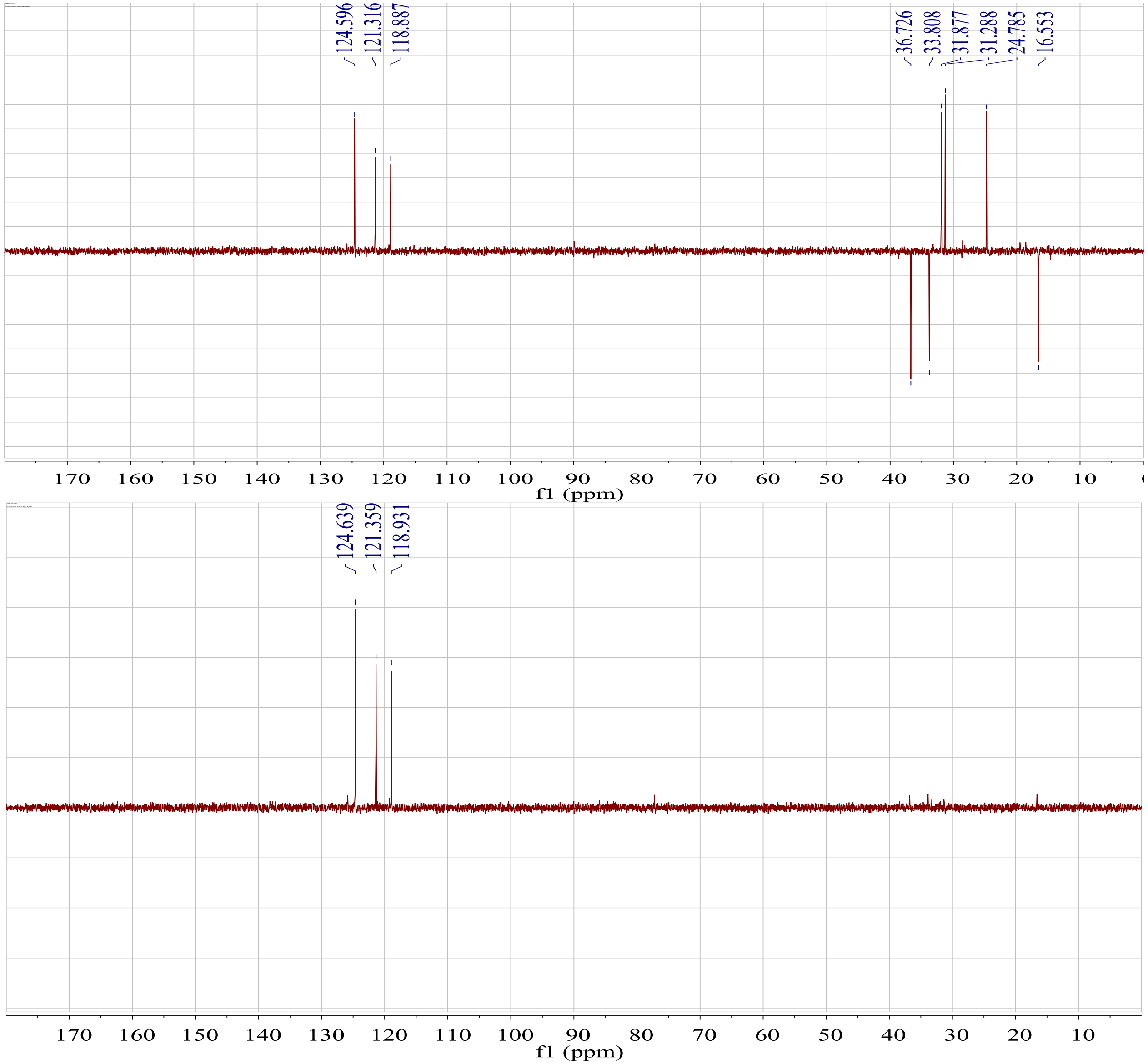

Figure S6. gHMQC of pseudaboydin A (**1**).

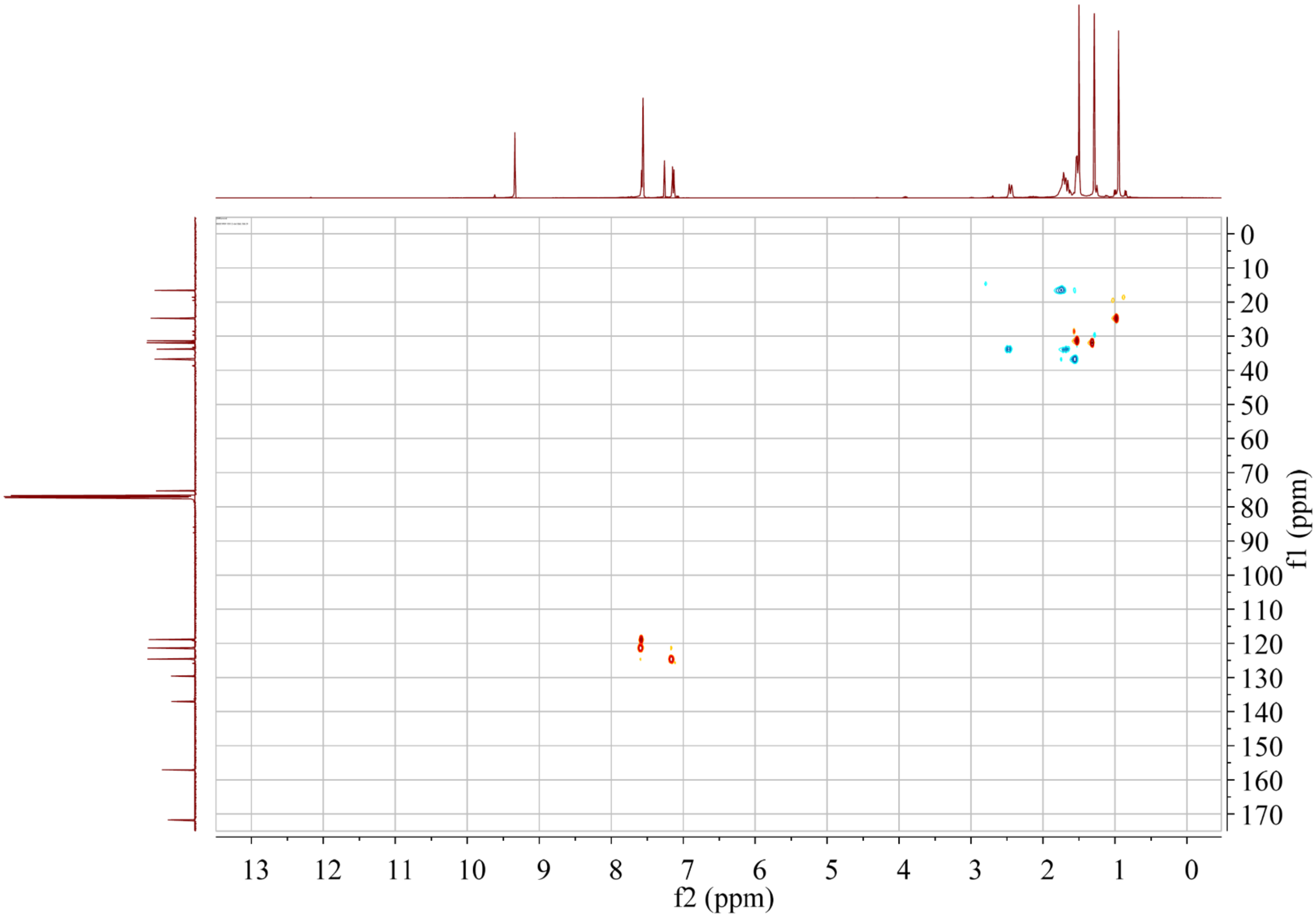

Figure S7. gHMBC of pseudaboydin A (**1**).

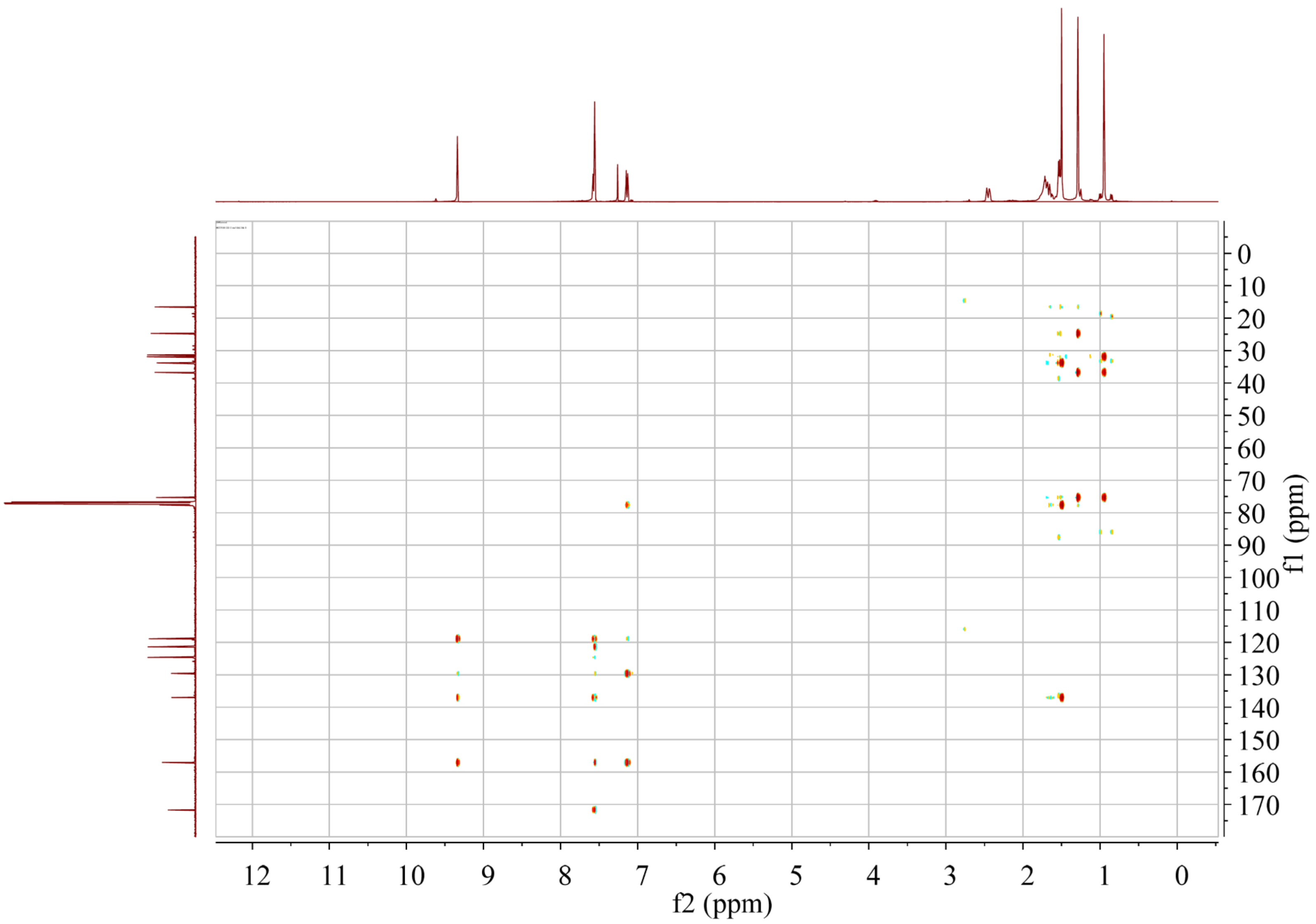

Figure S8. ^{1}H–^{1}H gCOSY of pseudaboydin A (**1**).

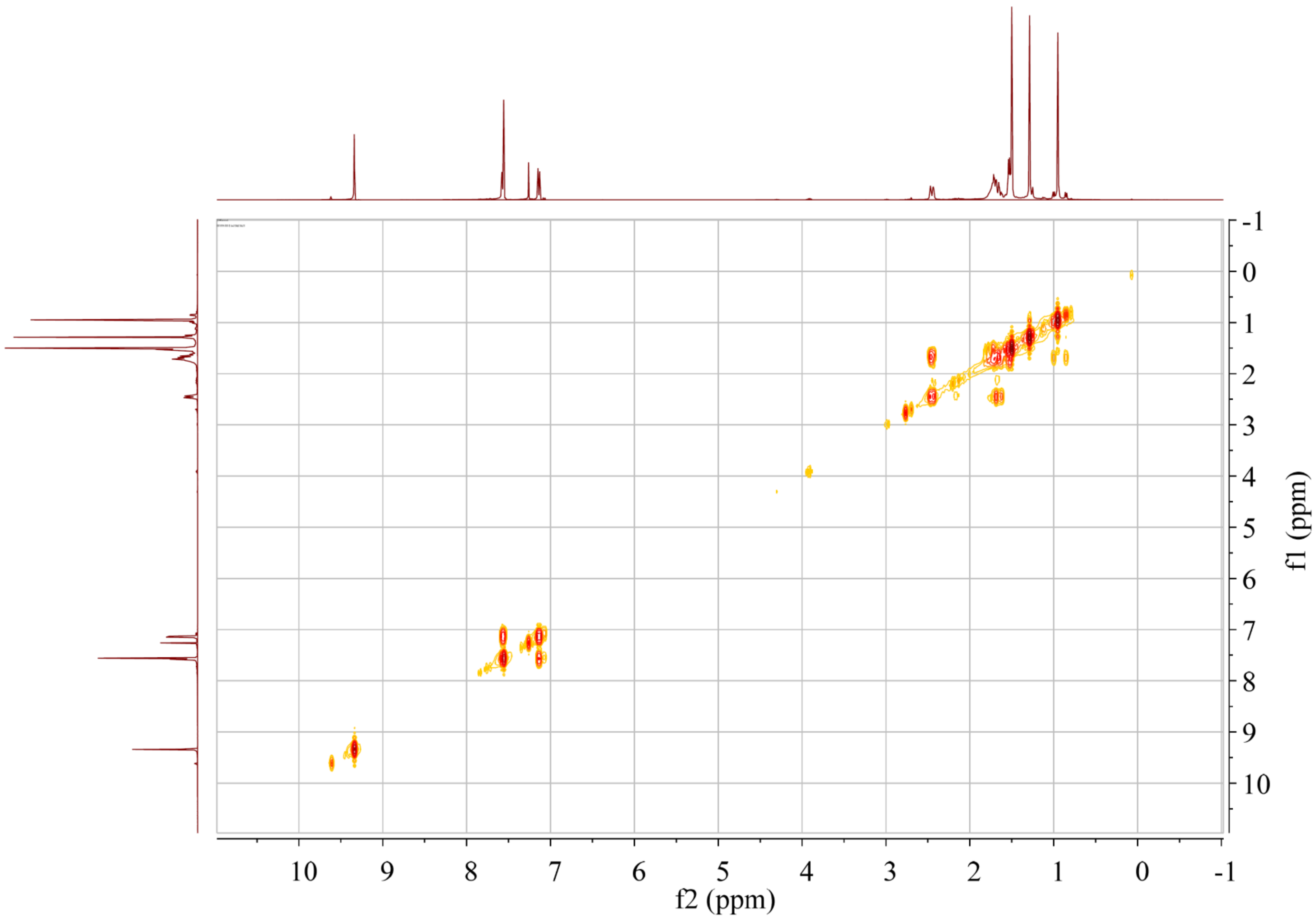

Figure S9. LREIMS of pseudaboydin B (**2**).

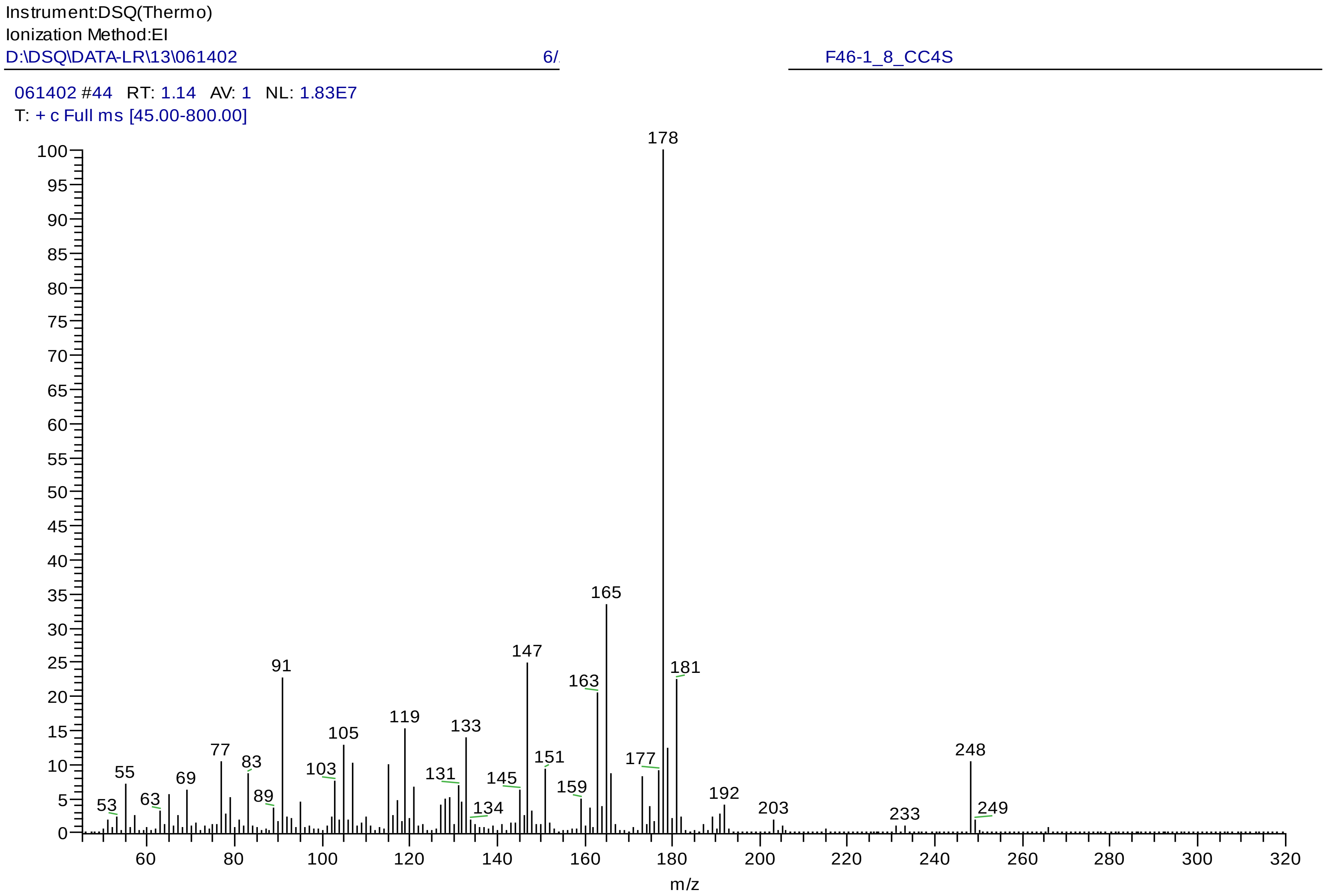

Figure S10. HREIMS of pseudaboydin B (**2**).

SPECTRUM - MS
File: D:\DATA-HR\13\082702-f46-c1.RAW
Full ms [228.500 - 256.500] - Range: 248.100 - 248.200
Scan No. 16 of 21
Scan #: 16
RT: 0.61
Data points: 1

Mass	Relative Intensity	Theoretical Mass	Delta[ppm]	Delta[mmu]	RDB	Composition
248.1408	87.8	248.1407	0.5	0.1	6.0	$C_{15} H_{20} O_3$

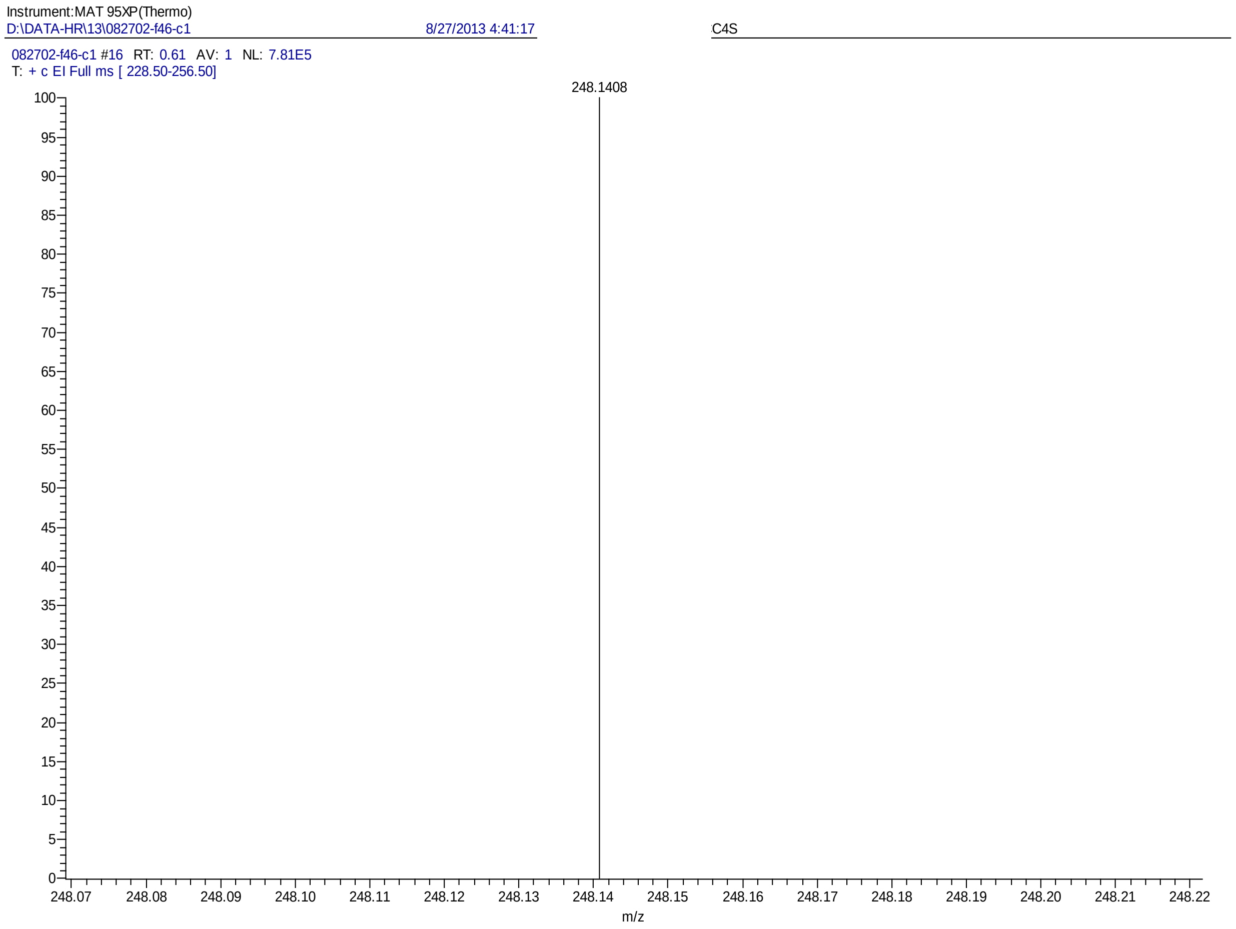

Figure S11. ^{1}H-NMR (400 MHz, $CDCl_3$) spectrum of pseudaboydin B (**2**).

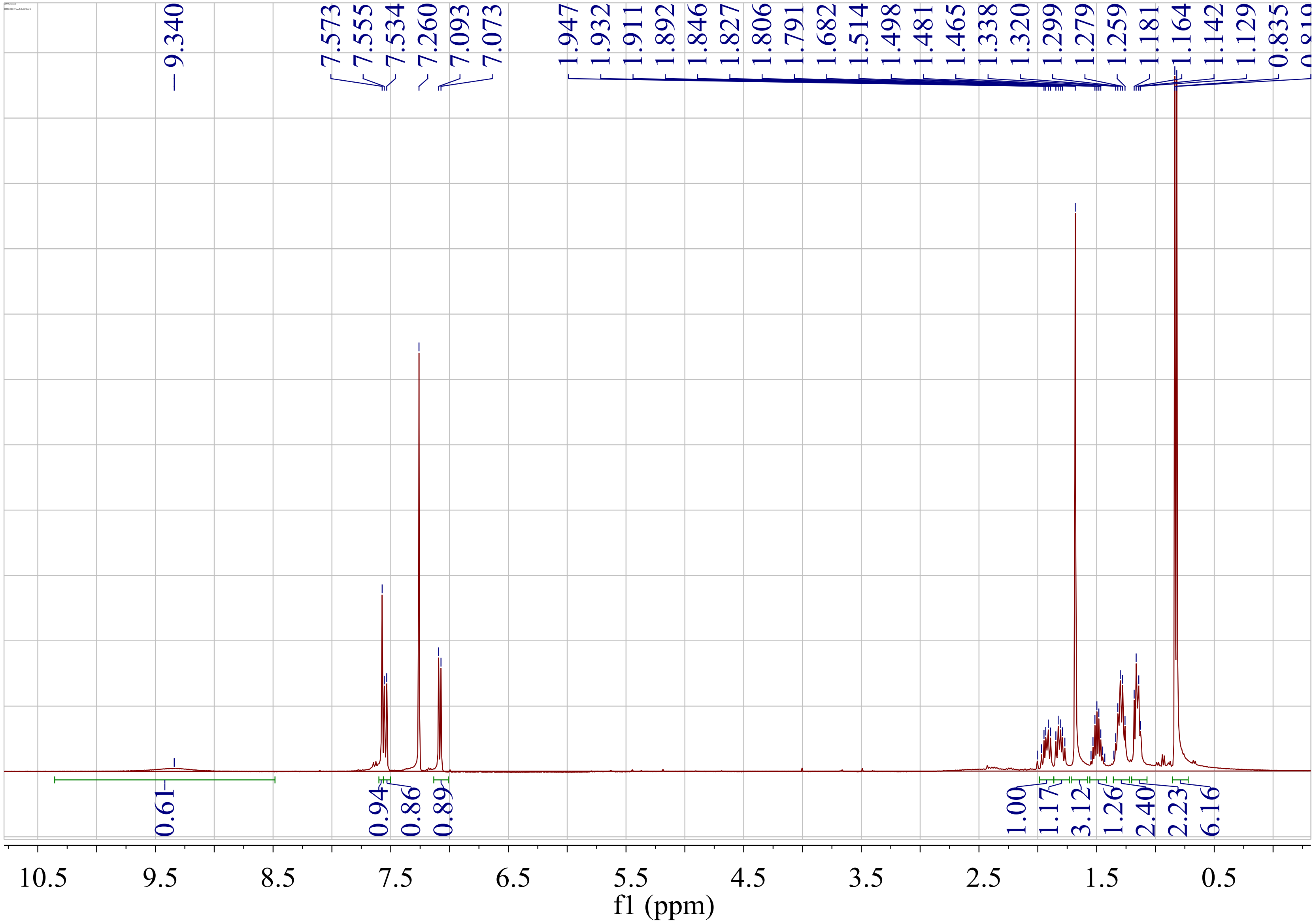

Figure S12. ^{13}C-NMR (100 MHz, $CDCl_3$) spectrum of pseudaboydin B (**2**).

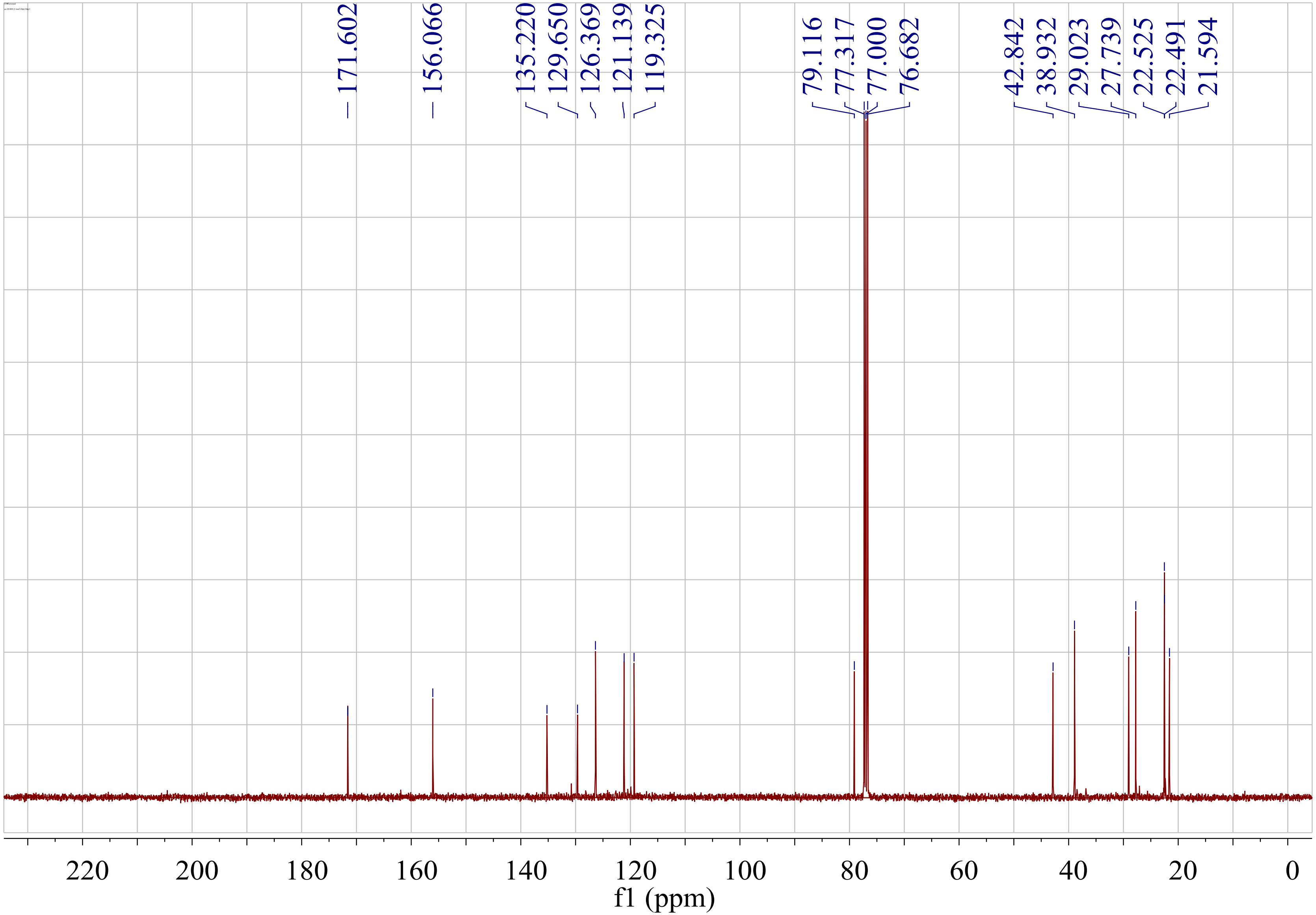

Figure S13. DEPT135 and DEPT90 of pseudaboydin B (**2**).

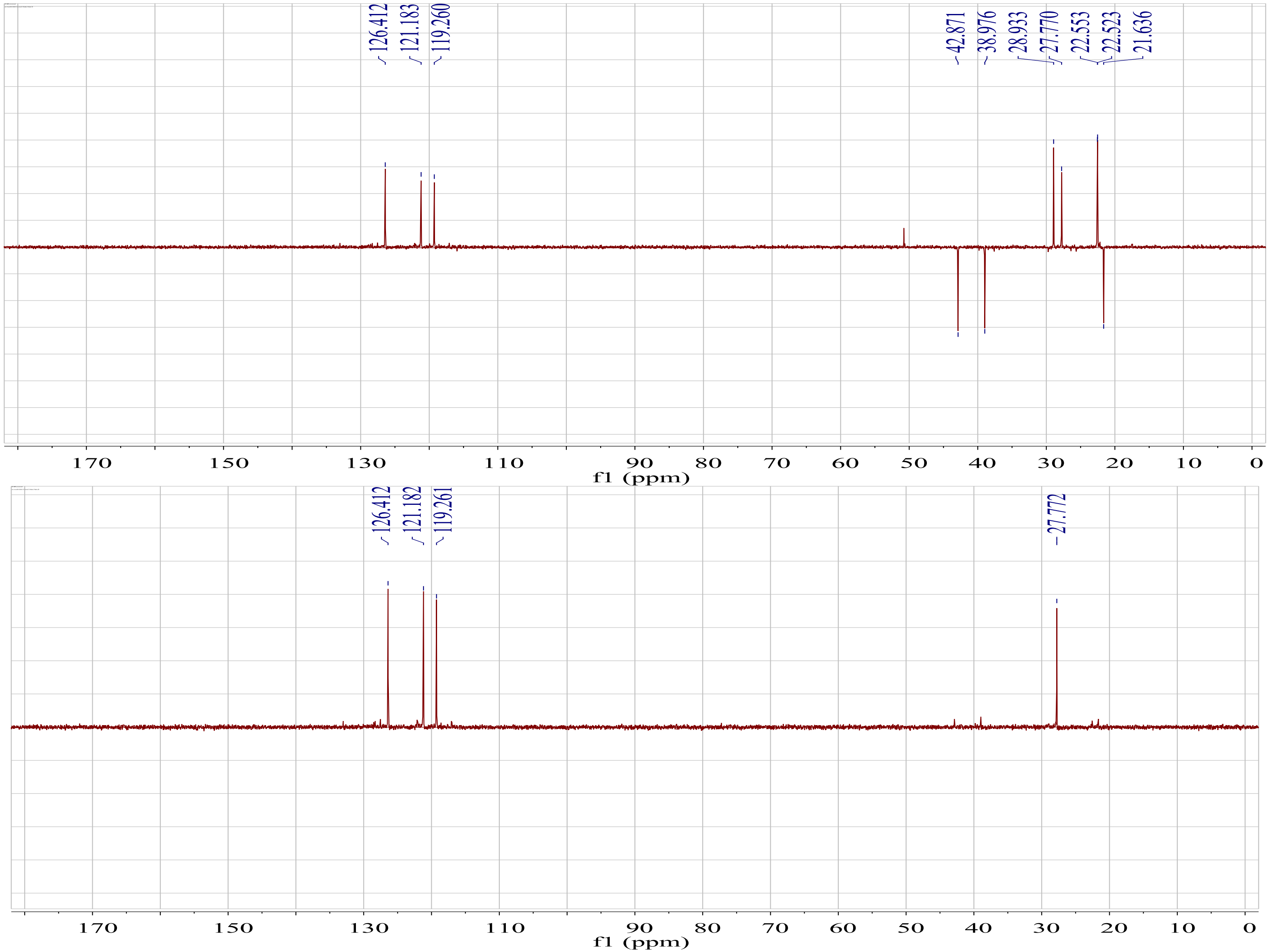

Figure S14. gHMQC of pseudaboydin B (**2**).

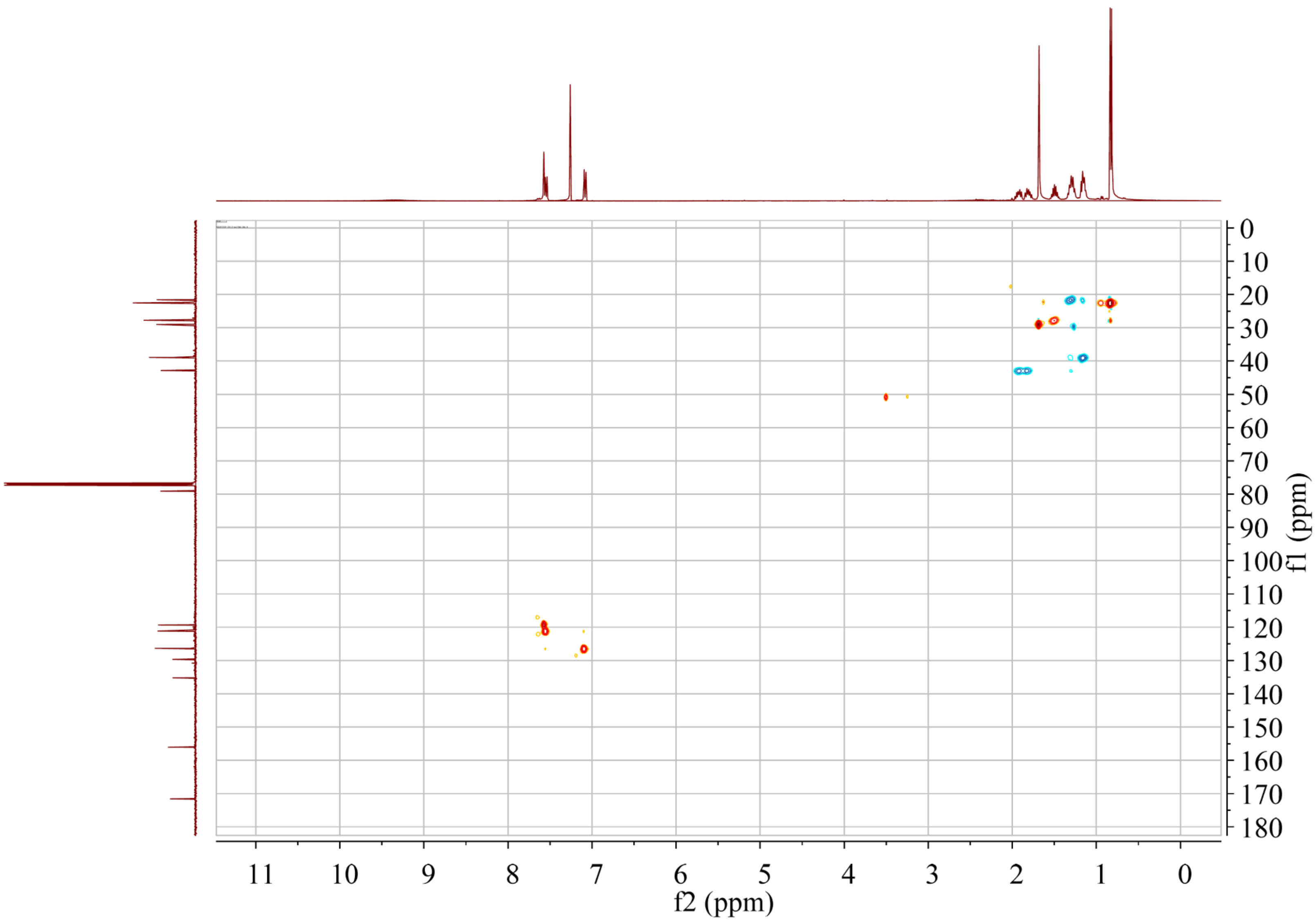

Figure S15. gHMBC of pseudaboydin B (**2**).

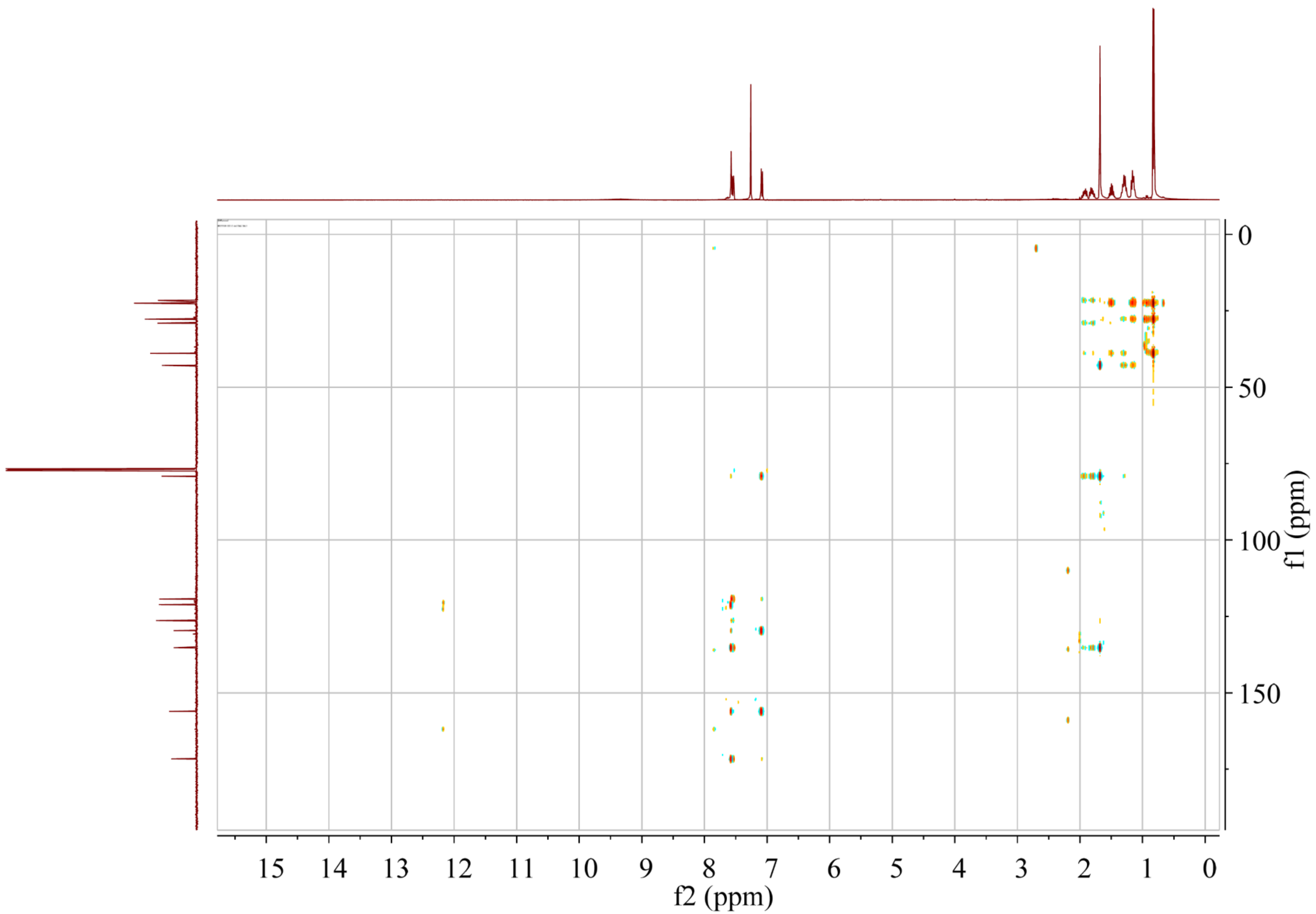

Figure S16. ^1H–^1H gCOSY of pseudaboydin B (**2**).

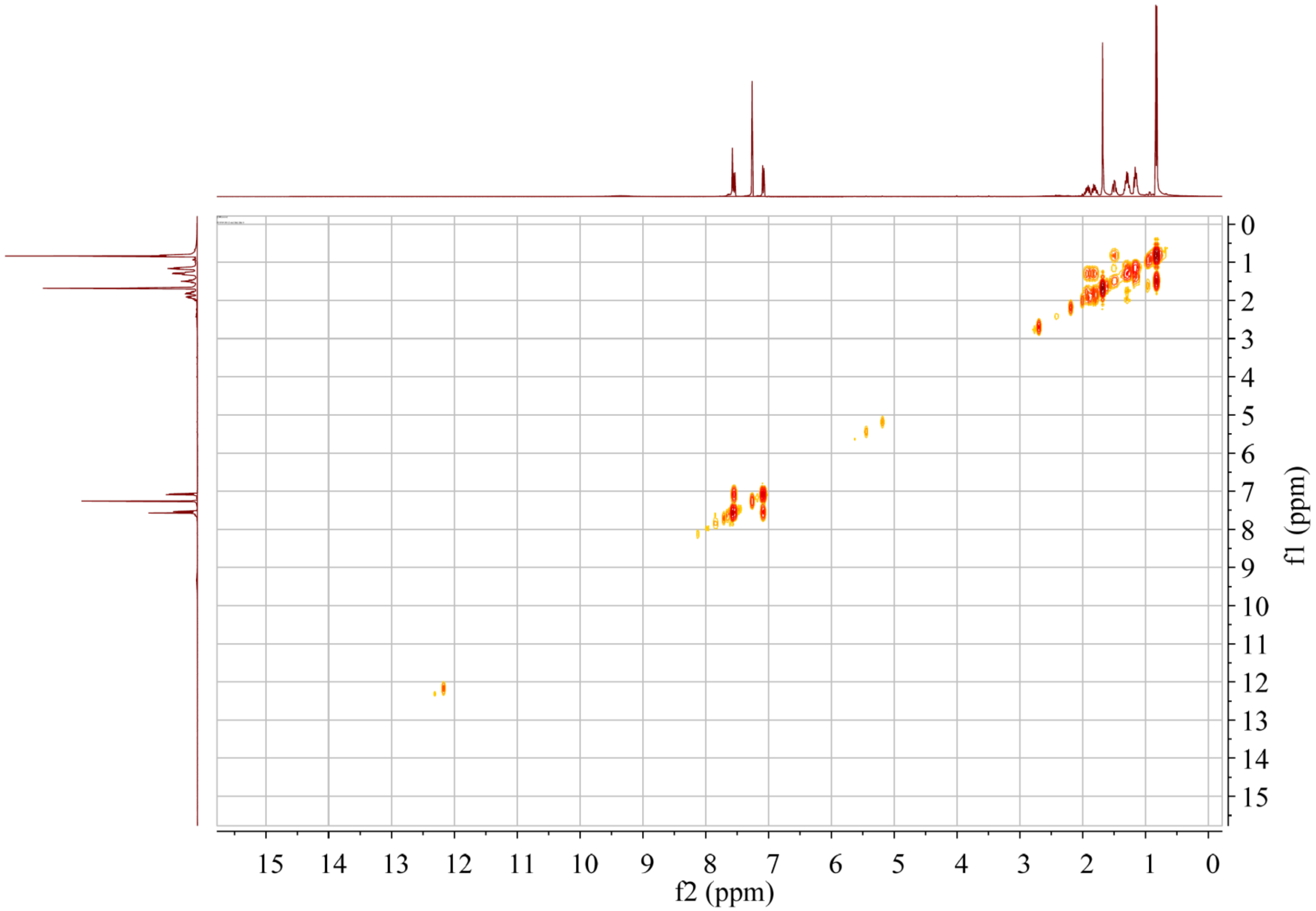

Figure S17. ^{1}H-NMR (400 MHz, DMSO-d6) spectrum of (*R*)-2-(2-hydroxypropan-2-yl)-2,3-dihydro-5-hydroxybenzofuran (**3**).

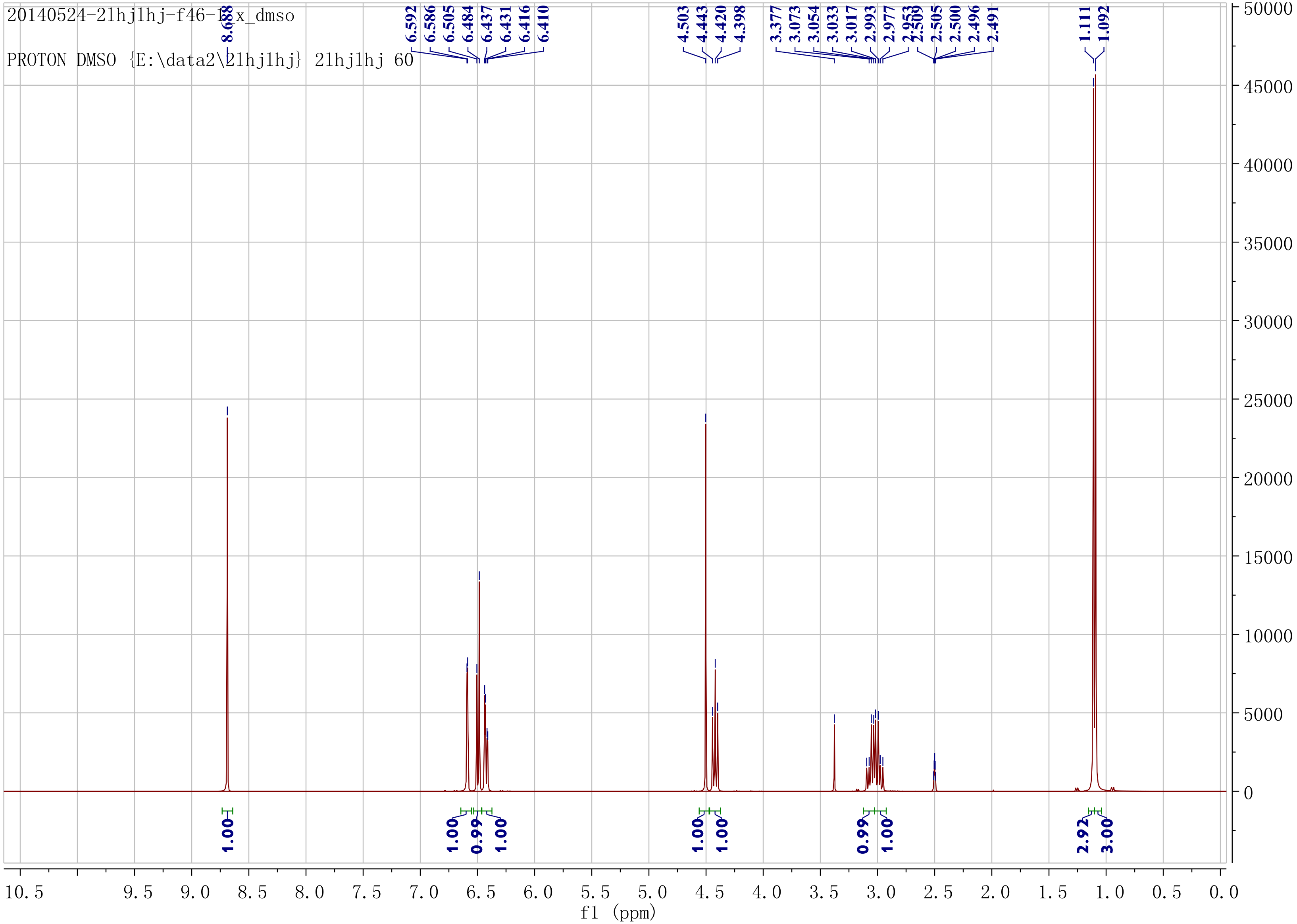

Figure S18. ^{13}C-NMR (100 MHz, DMSO-d6) spectrum of (*R*)-2-(2-hydroxypropan-2-yl)-2,3-dihydro-5-hydroxybenzofuran (**3**).

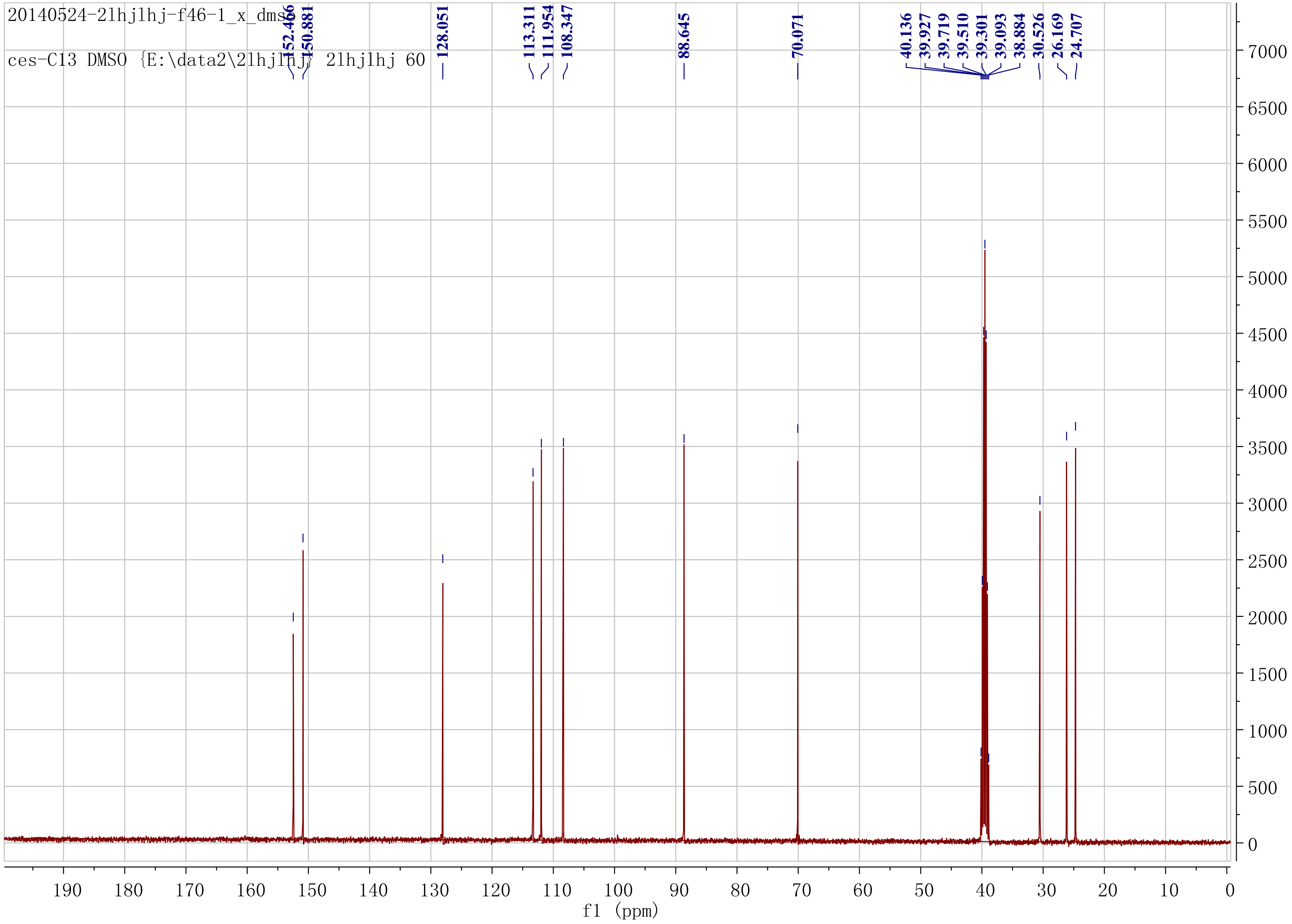

Figure S19. ^{1}H-NMR (400 MHz, $CDCl_3$) spectrum of (*R*)-2-(2-hydroxypropan-2-yl)-2,3-dihydro-5-methoxybenzofuran (**4**).

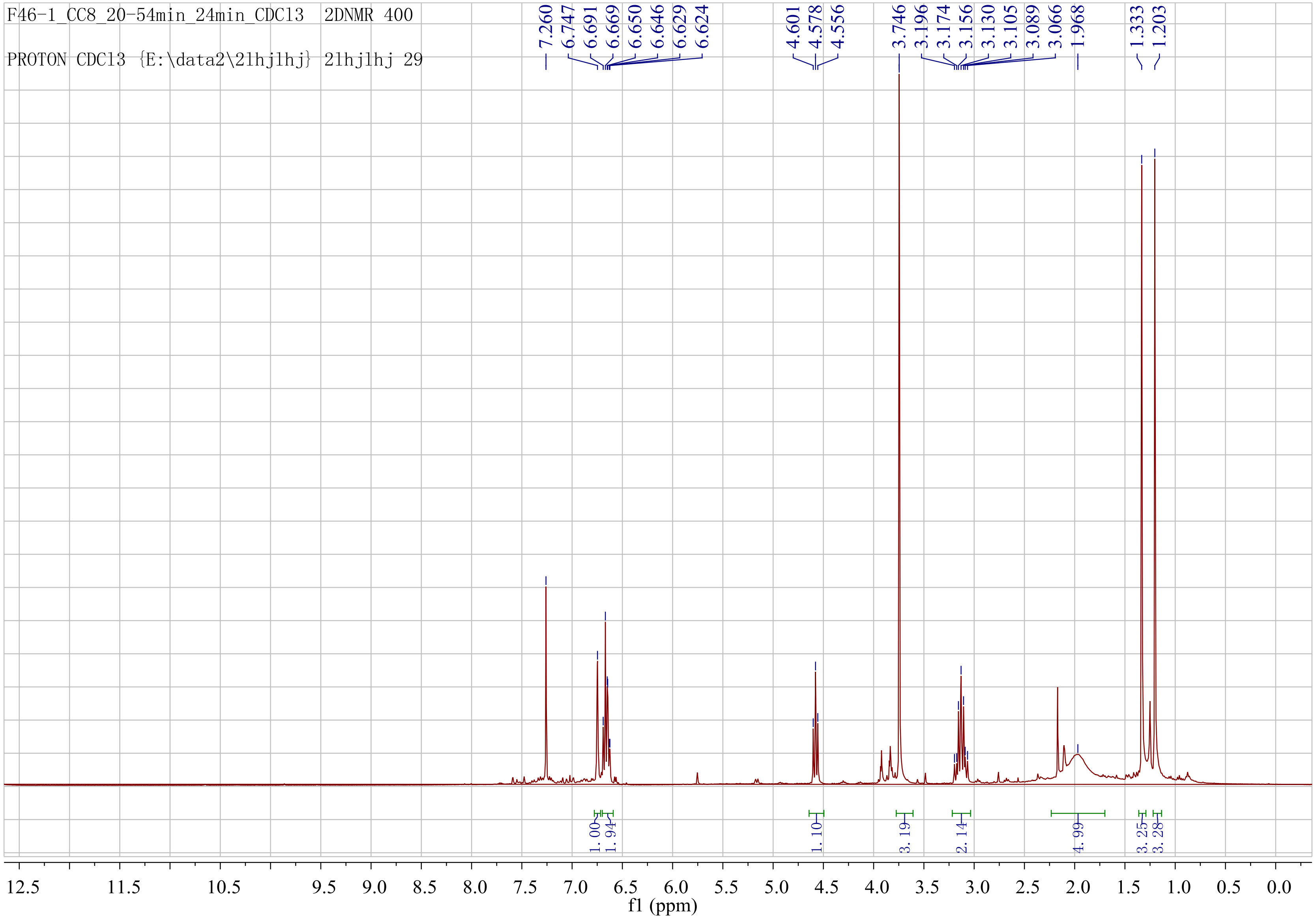

Figure S20. ^{13}C-NMR (100 MHz, $CDCl_3$) spectrum of (*R*)-2-(2-hydroxypropan-2-yl)-2,3-dihydro-5-methoxybenzofuran (**4**).

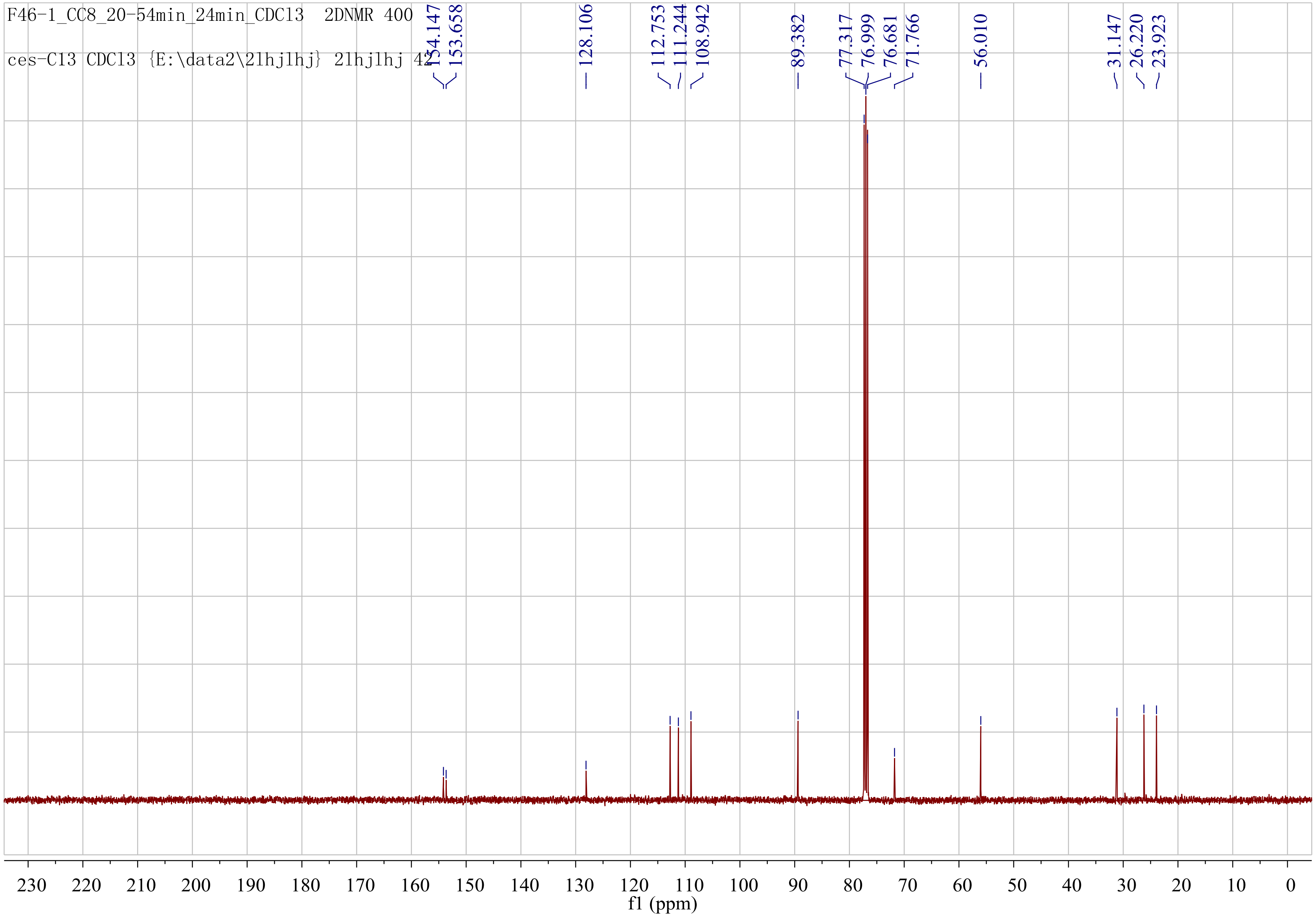

Figure S21. ^{1}H-NMR (400 MHz, $CDCl_3$) spectrum of 3,3′-dihydroxyl-5,5′-dimethyldiphenyl ether (**5**).

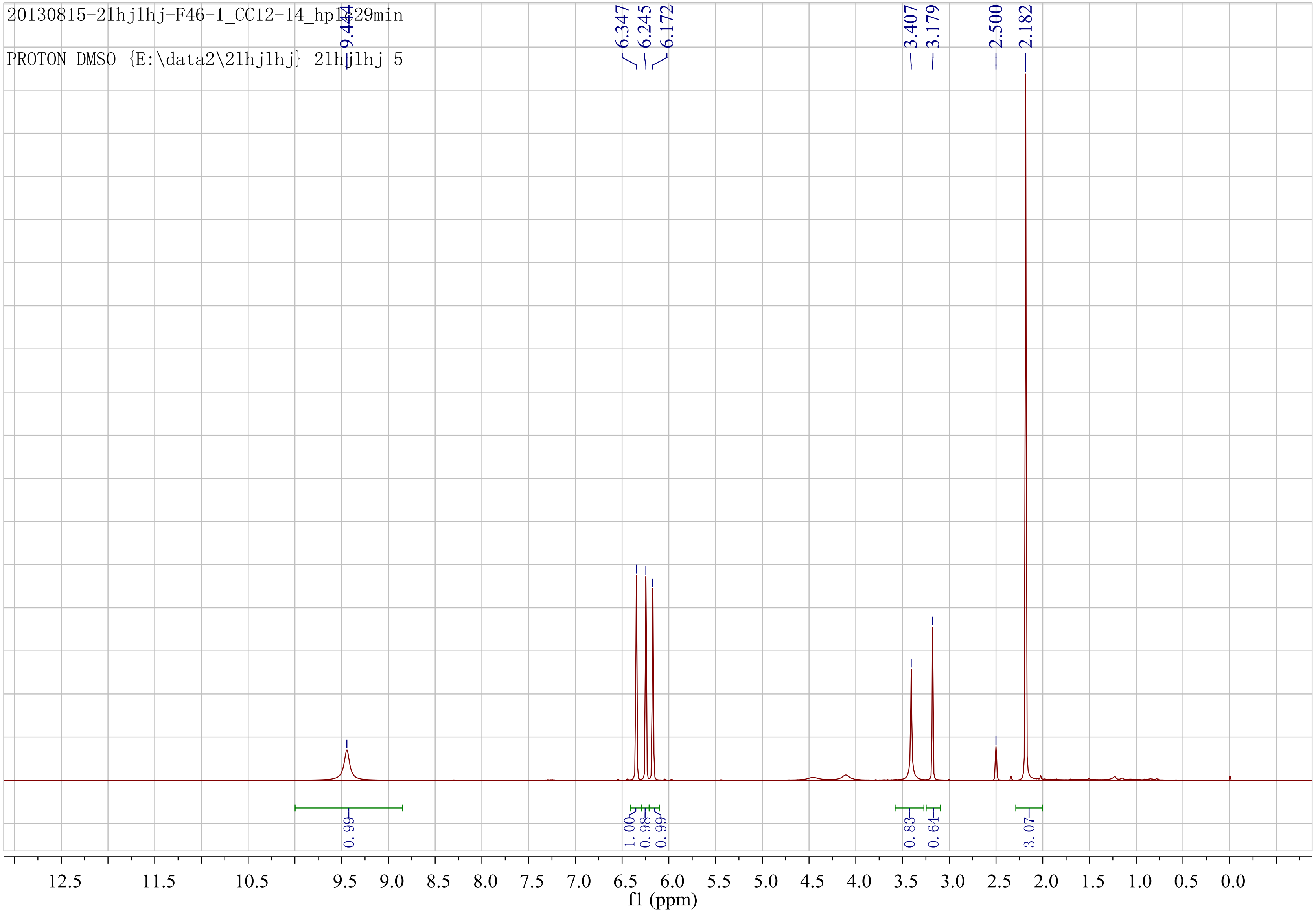

Figure S22. ^{13}C-NMR (100 MHz, $CDCl_3$) spectrum of 3,3′-dihydroxyl-5,5′-dimethyldiphenyl ether (**5**).

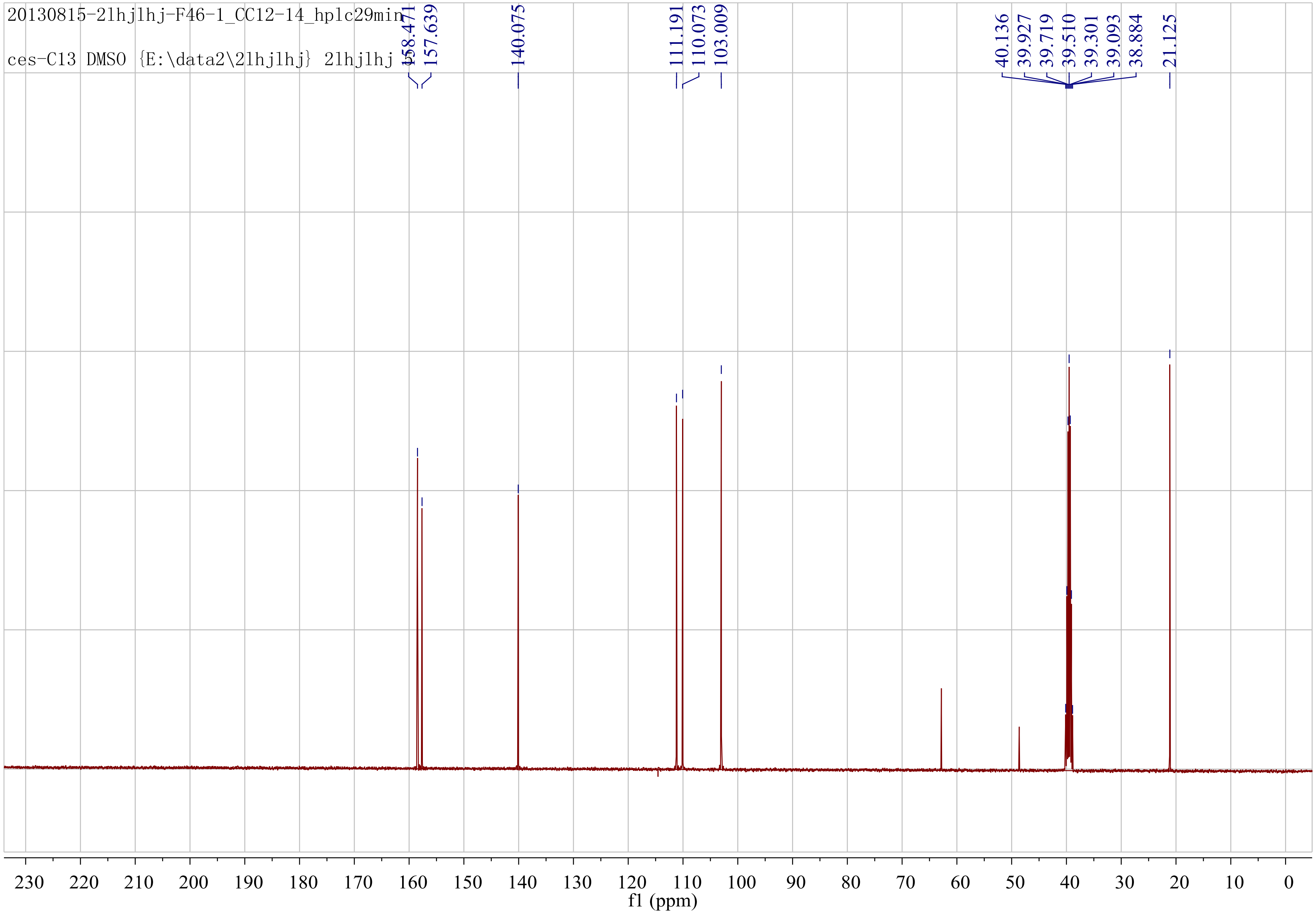

Figure S23. ^{1}H-NMR (400 MHz, $CDCl_3$) spectrum of 3-(3-methoxy-5-methylphenoxy)-5-methylphenol (**6**).

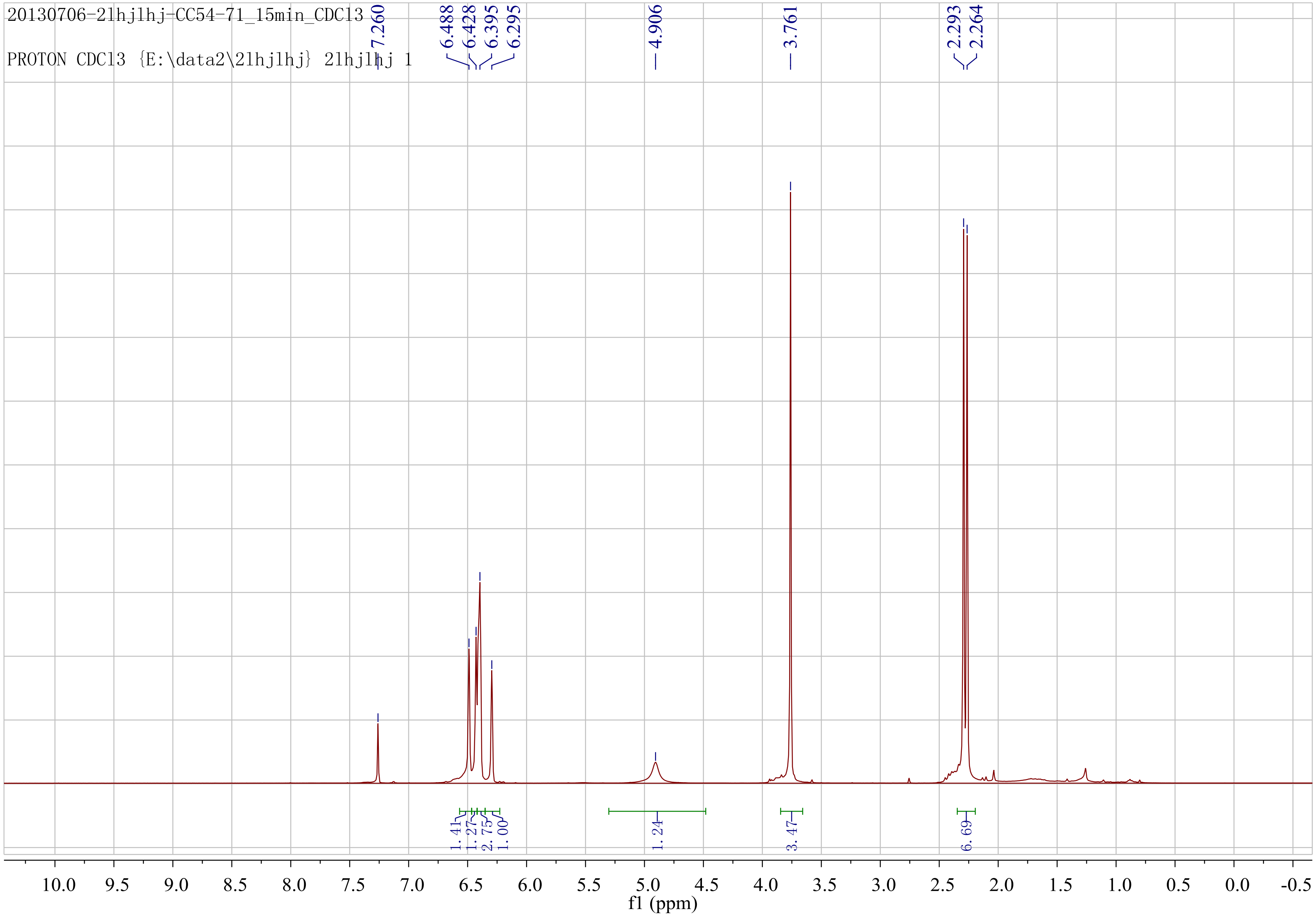

Figure S24. ^{13}C-NMR (100 MHz, $CDCl_3$) spectrum of 3-(3-methoxy-5-methylphenoxy)-5-methylphenol (**6**).

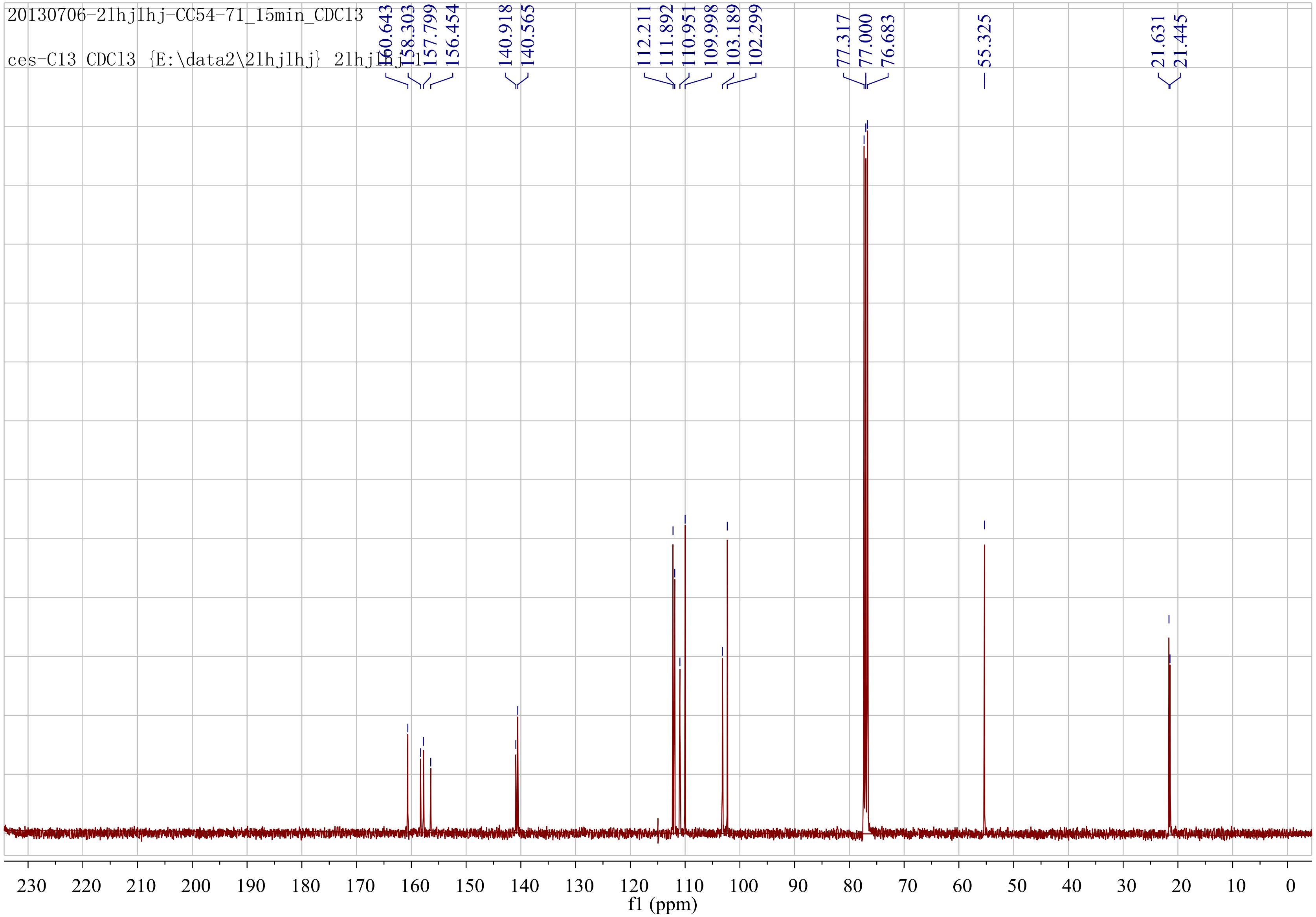

Figure S25. ^{1}H-NMR (400 MHz, CD_3OD) spectrum of 3-(3-methoxy-5-methylphenoxy)-5-methylphenol (**6**).

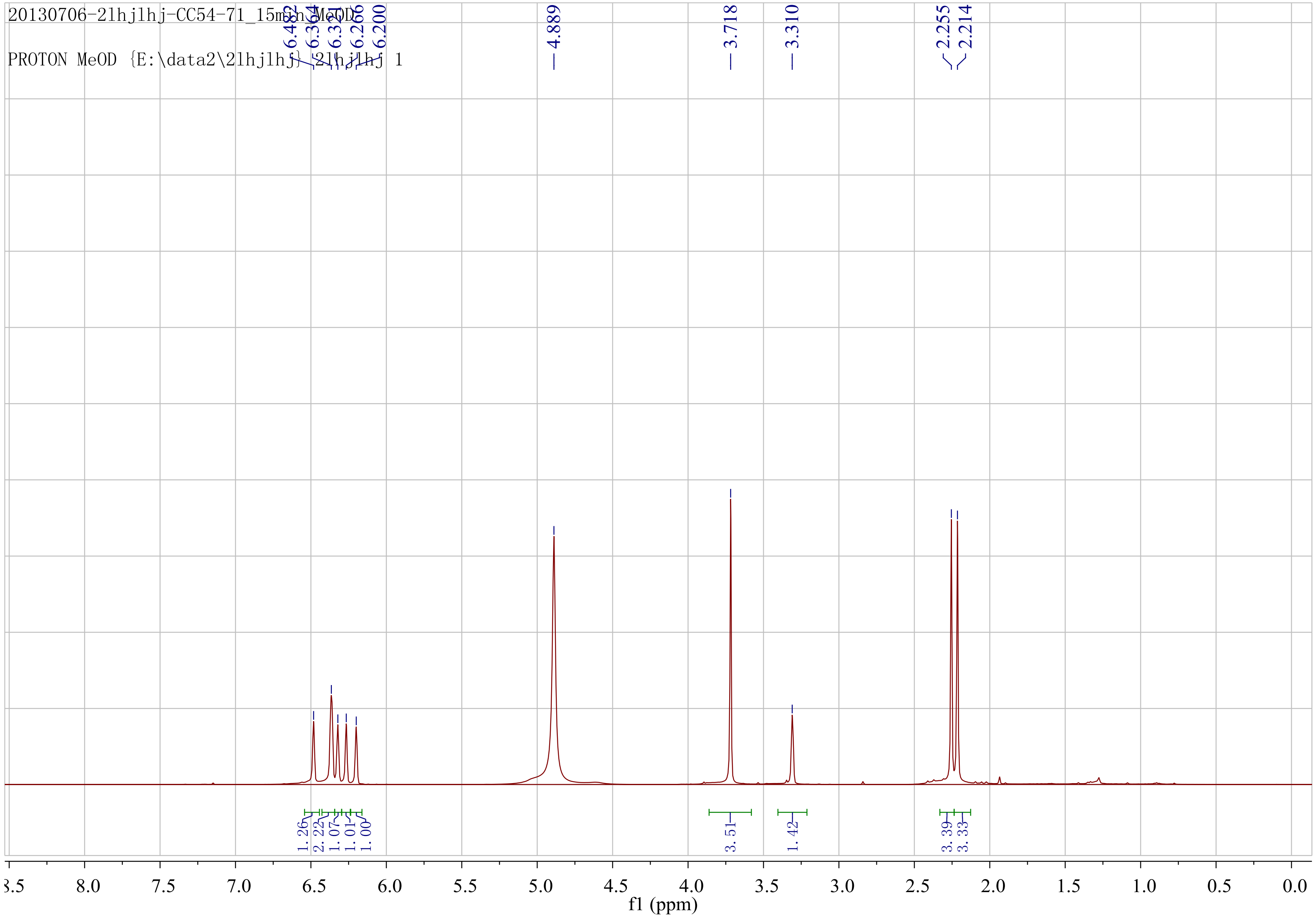

Figure S26. ^{13}C-NMR (100 MHz, CD_3OD) spectrum of 3-(3-methoxy-5-methylphenoxy)-5-methylphenol (**6**).

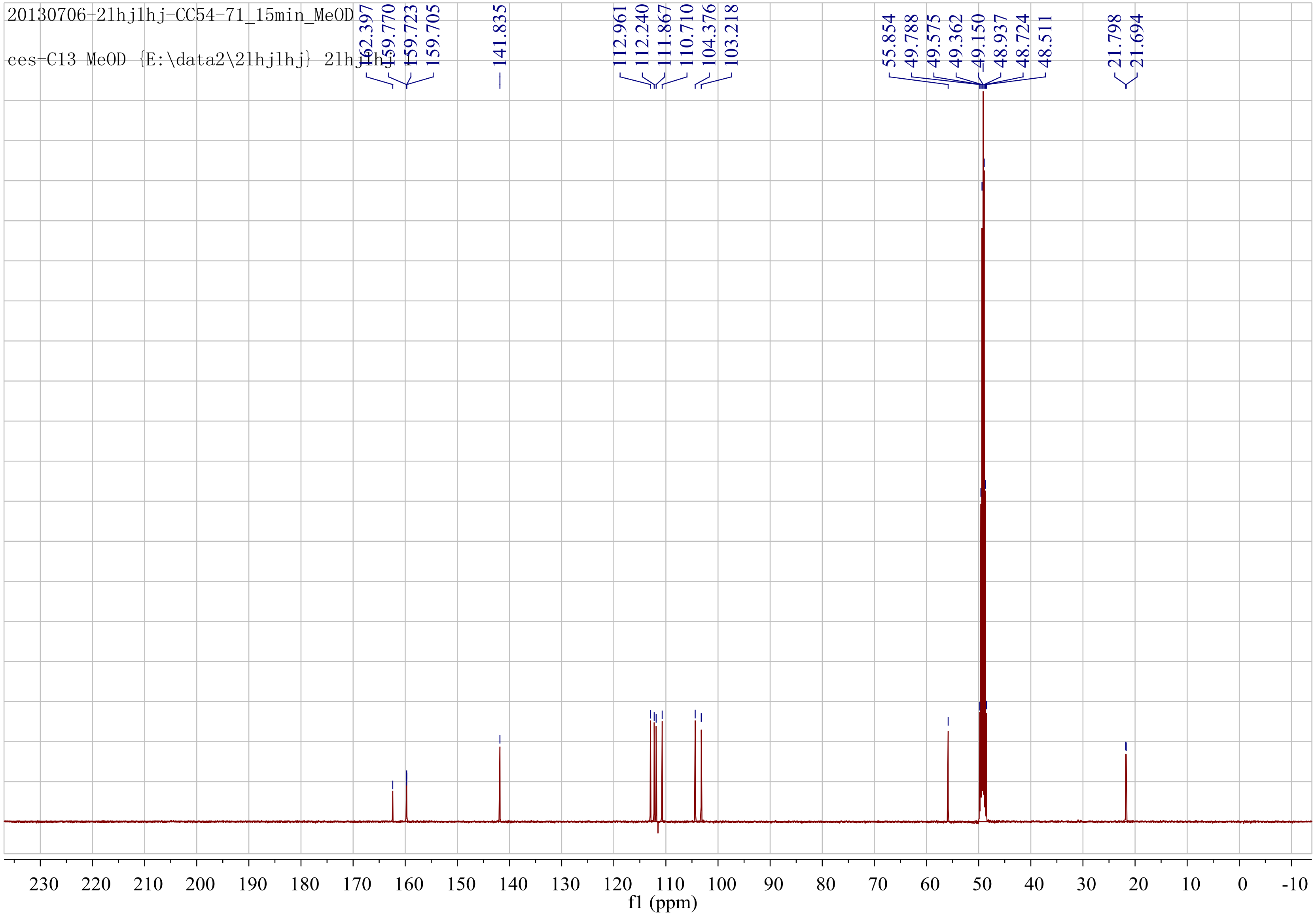

Figure S27. ^{1}H-NMR (400 MHz, $CDCl_3$) spectrum of (−)-regiolone (**7**).

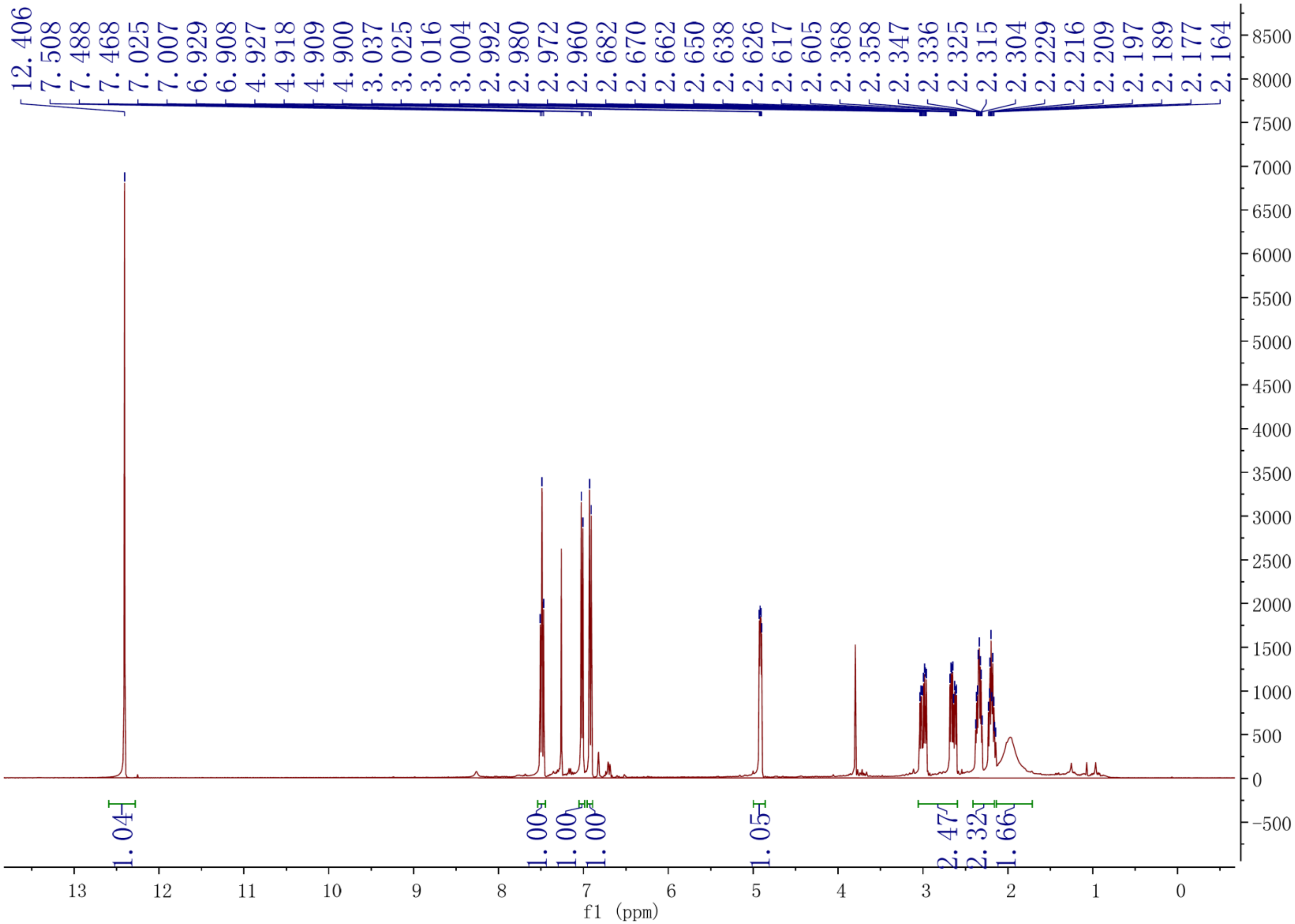

Figure S28. ^{13}C-NMR (100 MHz, $CDCl_3$) spectrum of (−)-regiolone (**7**).

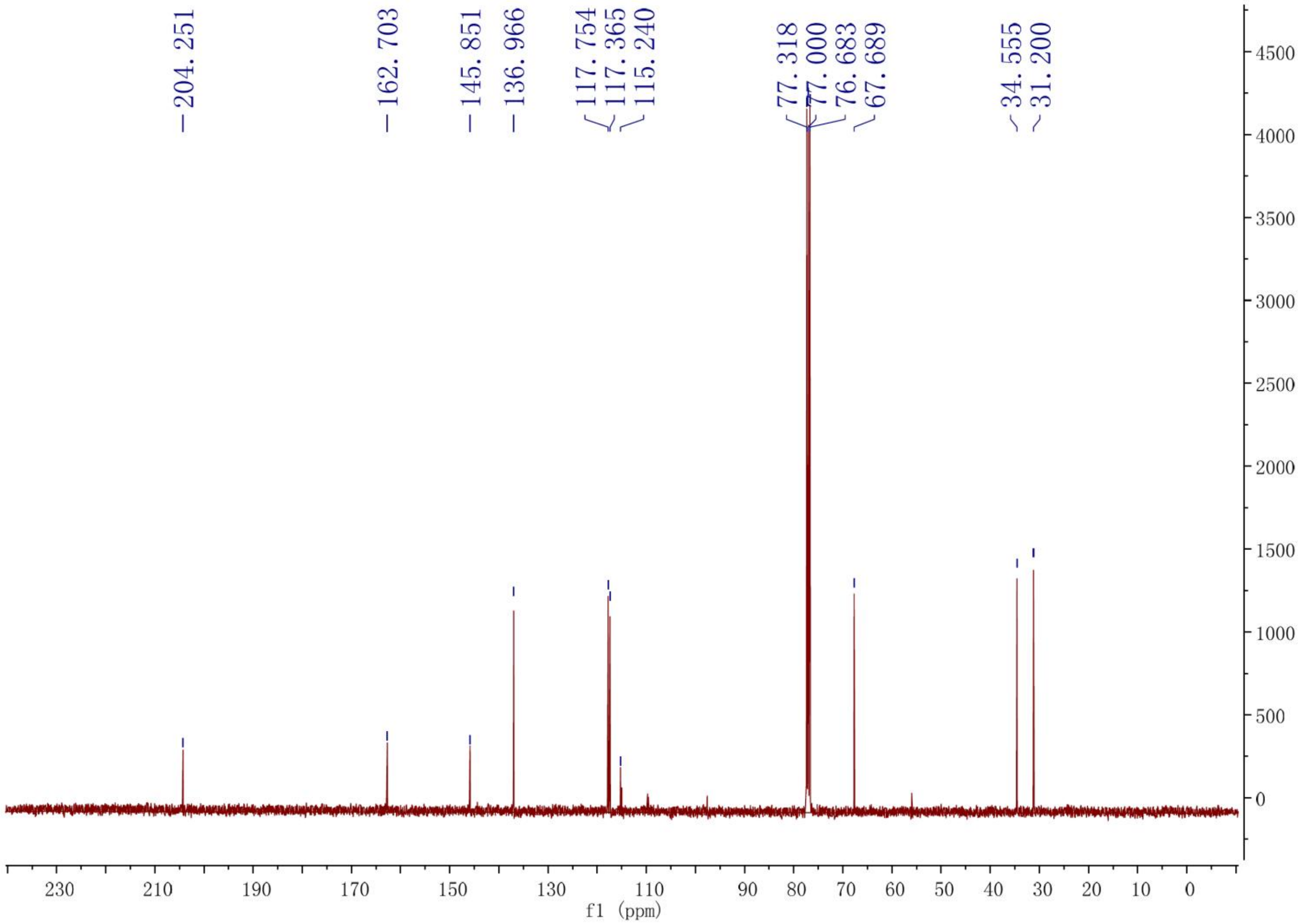

© 2014 by the authors; licensee MDPI, Basel, Switzerland. This article is an open access article distributed under the terms and conditions of the Creative Commons Attribution license (http://creativecommons.org/licenses/by/3.0/).
